# Three Novel Bacteriophages, J5a, F16Ba, and z1a, Specific for *Bacillus anthracis*, Define a New Clade of Historical Wbeta Phage Relatives

**DOI:** 10.3390/v14020213

**Published:** 2022-01-21

**Authors:** Aleksandra Nakonieczna, Paweł Rutyna, Magdalena Fedorowicz, Magdalena Kwiatek, Lidia Mizak, Małgorzata Łobocka

**Affiliations:** 1 Biological Threats Identification and Countermeasure Center, Military Institute of Hygiene and Epidemiology, 24-100 Pulawy, Poland; pawel.rutyna@wihe.pl (P.R.); magdalena.fedorowicz@wihe.pl (M.F.); magdalena.kwiatek@wihe.pl (M.K.); lidia.mizak@wihe.pl (L.M.); 2 Institute of Biochemistry and Biophysics of the Polish Academy of Sciences, 02-106 Warsaw, Poland

**Keywords:** *Bacillus anthracis*, bacteriophages, complete genomic sequence, siphovirus, *Wbetavirus*, endolysin, receptor-binding protein, arbitrium system, phage tail

## Abstract

*Bacillus anthracis* is a potent biowarfare agent, able to be highly lethal. The bacteria dwell in the soil of certain regions, as natural flora. Bacteriophages or their lytic enzymes, endolysins, may be an alternative for antibiotics and other antibacterials to fight this pathogen in infections and to minimize environmental contamination with anthrax endospores. Upon screening environmental samples from various regions in Poland, we isolated three new siphophages, J5a, F16Ba, and z1a, specific for *B. anthracis.* They represent new species related to historical anthrax phages Gamma, Cherry, and Fah, and to phage Wbeta of *Wbetavirus* genus. We show that the new phages and their closest relatives, phages Tavor_SA, Negev_SA, and Carmel_SA, form a separate clade of the *Wbetavirus* genus, designated as J5a clade. The most distinctive feature of J5a clade phages is their cell lysis module. While in the historical phages it encodes a canonical endolysin and a class III holin, in J5a clade phages it encodes an endolysin with a signal peptide and two putative holins. We present the basic characteristic of the isolated phages. Their comparative genomic analysis indicates that they encode two receptor-binding proteins, of which one may bind a sugar moiety of *B. anthracis* cell surface.

## 1. Introduction

Anthrax is a serious, often fatal zoonotic disease caused by *B. anthracis*, a spore-forming rod typically found in soil within the environment of herbivores (i.e., sheep, cattle, and goats) [1]. Spores are extremely resistant to harsh environmental conditions, and for this reason, they can survive in the soil for years and infect grazing animals [2]. Natural human infections occur sporadically in developed countries, mainly in people exposed to infected animal products (e.g., meat or hides).

*B. anthracis*, especially in the form of endospores, is one of the greatest biowarfare threats because of its high morbidity and mortality rates, low infective dose, relative ease of production, and difficulty in detection and decontamination. In the case of the intentional release of aerosolized spores or the contamination of drinking water, there is a high risk to national security and public health [3]. Currently, according to the CDC, the recommended procedure in case of anthrax infection relies on broad-spectrum antibiotics or antitoxin therapy. However, an antibiotic application can take up to 60 days and may include intravenous administration. Additionally, to have a chance of a full recovery, it is important to get medical care in a short time after exposure [4]. One may suspect that *B. anthracis* strains with naturally or artificially developed antibiotic resistance can be potentially used in a bioterrorist attack [5]. Therefore, it is of public interest to develop novel, rapid antibacterial agents against *B. anthracis*, with different modes of action than those of commonly used antibiotics. Phages and their lytic enzymes, endolysins (or lysins), may become such agents due to their high specificity and activity against antibiotic-resistant bacteria.

*B. anthracis* is a member of *Bacillus cereus* group. Its strains have been clustered based on genomic and physiological analysis to one of the three clades of this group [6,7]. Their feature commonly considered as differentiating and essential for pathogenicity is the production of two exotoxins (lethal toxin and edema toxin) and poly-γ-d-glutamic acid capsule [8]. The toxin and capsule are encoded by two separate plasmids, pXO1 and pXO2, respectively. However, strains of other *B. cereus* group species with these plasmids have been also isolated. Additionally, at the genomic level, *B. anthracis* is so similar to certain other *B. cereus* group species that it is believed to have evolved as a separate species only recently. Moreover, within species, *B. anthracis* strains show little genetic diversity, presumably as a result of long dormancy periods in the form of endospores in soil [9]. These features mean that the chances of finding novel specific *B. anthracis*-infecting phages are limited and that certain phages infecting *B. anthracis* can infect also *B. cereus* group strains.

Previously isolated phages infecting *B. cereus* group members display diversity in morphology and host range. They were classified in the past into the *Siphoviridae* (e.g., Wβ, Wip2, Wip4, and Frp2), *Myoviridae* (e.g., Wip5, Frp1, Crookii), or *Tectiviridae* (e.g., Wip1 and Htp1) families [10,11]. To date, whole genomes of over 404 *Bacillus* phages have been deposited in GenBank (http://www.ncbi.nlm.nih.gov, accessed on 30 November 2021). Sequence comparisons revealed that they belong to at least 12 highly diverse evolutionary groups [12]. Many of them have proven utility for typing or can be potentially useful in phage therapy or biocontrol [13]. The first *B. anthracis*-specific phage was isolated in 1930 from crude sewage [14]. Since then, several additional *B. anthracis* phages have been isolated, among them the well-known phage Gamma [15]—a diagnostic tool to distinguish *B. anthracis* from other *B. cereus* group members [16]. As a result of the genetic instability and the widespread use of Gamma phage, there are now several similar, yet genetically distinct, Gamma-like phages (e.g., temperate phage Wbeta and virulent phages Fah, LSU, USAMRIID, and Cherry) [17,18].

Particular attention is paid to *B. anthracis* phage lysins that cleave the peptidoglycan cell wall of infected bacteria to allow for the passage of infecting phage DNA or for the release of progeny virions [19]. For example, PlyG lysin, isolated from the Gamma phage, specifically kills both vegetative cells and germinating spores of *B. anthracis* isolates and other members of the *B. anthracis* “cluster” of bacilli [20]. Another lysin, PlyPH, acquired by the *B. anthracis* genome from a phage source, has a high degree of sequence identity of the putative C-terminal domain with PlyG, but presumably different catalytic action. Both enzymes share an identical range of activities, which may suggest that they may recognize and bind the same cell wall epitope [21].

In this paper, we describe three new phages specific against *B. anthracis*. They can infect and lyse only *B. anthracis* and none of the other *B. cereus* group strains tested. The new phages show only around 69–87% identity to certain other anthrax phages. Nucleotide sequences of their endolysins show noticeable differences from those encoded by Gamma, Cherry, or Fah phages. The newly isolated phages could potentially find use in certain applications, such as in *B. anthracis* identification and detection assays [2]. Their engineered derivatives depleted of genomic modules essential for lysogeny might be also useful in treating human or animal infections (likewise their endolysins), or as decontaminants or disinfectants (of skin, surface, or clothes).

## 2. Materials and Methods

### 2.1. Bacterial Strains

A complete list of the bacterial strains used in these studies is included in Table S1. The strains were used to determine the host range of the studied phages and they comprise bacteria from the *B. cereus* group and *B. subtilis* ATCC 6633 strain. The list includes transitional strains from the *B. cereus* group (*B. cereus* and/or *B. thuringiensis*) that do not have plasmids but have a chromosomal anthrax marker gene—Ba 813 [22]. Their origin is not confirmed, but presumably they may have originated from *B. anthracis* [23].

The vaccine strain of *B. anthracis* 34F2 was used as a phage host strain in all experiments. A blood Columbia agar, TSA, and TSB media were used to grow and culture the strains.

### 2.2. Isolation of the B. anthracis-Infecting Bacteriophages and Their Host Range Determination

Bacteriophages were isolated from environmental samples, e.g., soil and manure, collected in different regions of Poland (J5a—south-eastern Poland, Subcarpathia; F16Ba—north-eastern Poland, Podlasie region; z1a—central-eastern Poland, the Lublin Region). For this purpose, environmental samples (~10 g) topped up to 35 mL with TSB medium supplemented with MgSO_4_ (0.2%), were incubated overnight at 37 °C, with the addition of 100 µL of an overnight culture of *B. anthracis* 34F2 strain. The following day, the samples were treated with 0.5 mL chloroform, put on a shaker for 30 min, and centrifuged for 30 min at 4200× *g*. The supernatant was then syringe-filtered (PES 0.22 µm Millipore filter, Merck Millipore, Burlington, MA, USA), and 15 µL of the filtrate was spotted on a lawn of *B. anthracis* cells in a top layer (0.7% agar) of double-layered TSA Petri dish and incubated overnight at 37 °C. Clear plaques obtained the following day were picked, suspended in 1 mL of TM buffer (50 mM Tris-Cl, 10 mM MgSO_4_, pH 7.5), vortexed vigorously, and plated again on the top layer of TSA agar with the indicator *B. anthracis* cells on the double-layered TSA Petri dish (this plating method was used in all further experiments). This was repeated at least two more times to obtain a pure clone of the given phage. The individual phages were propagated on the host strain, syringe-filtered (0.22 µm), and the lysates were used in the following studies.

To determine the phage host range, the 10–15 µL aliquots of undiluted and diluted lysates containing a given phage were spotted on lawns of different *B. cereus* group bacteria in the TSA soft agar on the double-layered TSA Petri dish, and incubated overnight at 37 °C. The phage sensitivity of bacteria was assessed the next day based on the presence of clear lysis zones and plaques.

### 2.3. Pulsed-Field Gel Electrophoresis (PFGE)

PFGE was performed to estimate phage genome sizes and phage preparation purity. For this purpose, 60 µL of lysate containing phages was warmed to 54 °C and mixed with 60 µL of a pre-warmed, 2% plug agarose (CleanCut, Bio-Rad, Hercules, CA, USA), dispensed into plug molds, and solidified. The plugs were then incubated overnight at 54 °C with shaking, in 5 mL of PL buffer (Phage Lysis buffer—50 mM Tris, 50 mM EDTA, 1% SDS) with 25 µL of Proteinase K (20 mg/mL). The following day, the plugs were washed 3 times in 5 mL of a pre-warmed TE buffer (10 mM Tris, 1 mM EDTA, pH 8) for 15 min each time.

PFGE was performed in 1% pulsed-field certified agarose gel (Bio-Rad), at a voltage of 180 V and 6 V/cm, using a CHEF-DR III system (Bio-Rad). The separation was performed in 0.5× TBE buffer for 16 h at 4 °C. Following electrophoresis, the gel was stained in ethidium bromide (0.5 µg/mL) for 30 min.

### 2.4. Transmission Electron Microscopy

Lysates containing phages (17 mL each) were ultracentrifuged at 116,200× *g* for 2 h. The pellet was suspended in 0.1 M ammonium acetate (pH 7.5), centrifuged again (36,440× *g* for 2 h at 4 °C), and resuspended in 50 µL of 0.1 M ammonium acetate. The concentrated phage suspension (2.5 µL) was spotted on a 300 × 300-mesh Formvar/carbon-coated copper grid (TAAB Laboratories Equipment Ltd, Berks, England) and left for 3 min. Excess solution was removed with filter paper, and the grids were negatively stained with 2% uranyl acetate (pH 6.8–7) for 2–3 min. After removing the excess staining solution, the grids were dried for 15 min at room temperature. The samples were visualized in a JEM 1400 TEM microscope (JEOL Co., Tokyo, Japan) at 300,000× magnification.

### 2.5. Optimal Multiplicity of Infection Assay

To determine the optimal multiplicity of infection (MOI) for phage propagation, phage and bacteria titers were equaled, then mixed together at the following ratios: 1, 0.5, 0.1, 0.05, and 0.01, and incubated for 15 min at 37 °C. After the incubation, samples were centrifuged at 6100× *g* for 10 min, and the supernatant containing unabsorbed phages was discarded. The pellets were suspended in 100 µL of LB medium and incubated for 4 h at 37 °C with shaking to allow for the development of phages in infected cells. Then, the aliquots of serially diluted and undiluted suspensions were plated on double-layered Petri dishes with indicator bacteria, as described in Section 2.2. The plates were incubated overnight at 37 °C and served to count plaques. The phage-to-bacteria ratio at which the number of plaques obtained was the highest (optimal MOI) was used in further experiments.

### 2.6. Adsorption Efficiency Assay and One-Step Growth

A *B. anthracis* overnight culture in TSB medium was refreshed by 100× dilution and incubated with shaking at 37 °C until the optical density at 600 nm (OD_600_) reached 0.5 (approx. 6.5 × 10^6^ CFU/mL). After incubation, 10 mL of the bacterial culture was centrifuged at 3000× *g* for 10 min at 4 °C. The supernatant was removed, and the pellet was resuspended in 1 mL of LB medium. Phage stock was then mixed with the host bacteria at MOI 0.5 and incubated at 37 °C for 20 min, followed by centrifugation at 5000× *g* for 10 min at 4 °C. The supernatant with free phages was discarded, and the pellet was resuspended in 1 mL of a fresh LB medium. This step was repeated twice. After centrifugation, the pellet was suspended in 25 mL of a pre-warmed LB medium, and incubation at 37 °C with shaking (100 rpm) was started. The samples of supernatant (100 µL) were taken out of the suspension at every 5 or 10 min (including time zero), serially diluted, and used for plaque assay as described in Section 2.2.

For the determination of the adsorption efficiency of each phage to bacteria, a protocol similar to that described by Baptista et al. [24] for *Bacillus* phage SPP1 was adopted. An overnight culture of *B. anthracis* was refreshed by 100× dilution and cultured to OD_600_ ≤ 0.5 at 37 °C with constant shaking. The phage stock was mixed with bacteria at MOI 0.5 and vortexed. A sample of the mixture (100 µL, time zero) was immediately removed and diluted 10 times by transferring to a tube containing 0.9 mL of ice-cold LB. The rest of the mixture was incubated at 30 °C, and samples (100 µL) were taken every 5 min for up to 40 min, vortexed, mixed with 0.9 mL of ice-cold LB, and kept in ice. The tubes collected in an ice cooler were vortexed and centrifuged at 12,000× *g* for 5 min. Top samples (100 µL) of supernatants served to prepare serial dilutions, which were used for plaque assays on the double-layered TSA Petri dish to determine the concentration of unabsorbed phages remaining in the supernatant at each time point.

### 2.7. Sensitivity of Phages to Temperature and pH

The thermal stability of the phages was studied at five different temperatures (20, 37, 50, 60, and 70 °C). Phage titers were equaled, and 1 mL aliquots of the lysates (10^8^ PFU/mL in a TM buffer) were incubated at the respective temperatures for 10 min, 30 min, 60 min, and 3 h. Each time, the withdrawn volumes (100 µL) were serially diluted in a TM buffer and then plated using the double-layered TSA plates with indicator bacteria.

Phage sensitivity to pH was evaluated using a TM buffer adjusted with 1 M NaOH or HCl to the following pH values: 2, 3, 4, 6, 8, 10, 12, and 13. Aliquots of 500 µL of lysates (10^8^ PFU/mL in a TM buffer) were suspended in 4.5 mL of the respective buffer in sterile tubes. Tubes were incubated at room temperature for 1 h, then the suspensions were serially diluted and plated on double-layered Petri dishes.

### 2.8. Genomic DNA Extraction, Sequencing and Identification of Virion DNA Termini

Genomic DNA of the purified phages was extracted using the Genomic Mini AX Phage kit (A&A Biotechnology, Gdańsk, Poland) and digested with various restriction endonucleases to differentiate. The DNA of selected phages was sequenced.

Whole-genome sequencing and the initial assembly were performed at the Laboratory of DNA Sequencing and Oligonucleotide Synthesis of the Institute of Biochemistry and Biophysics in Warsaw. Genomic fragment libraries were constructed using a Kapa Library Preparation kit (KAPA/Roche), following phage DNA fragmentation using nebulization. The sequencing was performed using MiSeq (Illumina, San Diego, CA, USA). The raw sequence reads generated were processed using the cutadapt tool (https://journal.embnet.org/index.php/embnetjournal/article/view/200, accessed on 10 February 2018) to remove the remaining adapters and the FastX toolkit (http://hannonlab.cshl.edu/fastx_toolkit/, accessed on 10 February 2018) to delete low-quality data. Filtered data were assembled in contigs using Newbler v3.0 (Roche, Basel, Switzerland).

The assembly patterns of genomic sequence reads and PhageTerm software [25] were used to predict the termini of virion DNA molecules and DNA packaging mechanisms. To test whether the new phages contain single-stranded cohesive ends (*cos* sequence), their virion DNA molecules were treated with T4 DNA ligase and used as templates for PCR amplifications. For J5a and F16Ba phages, two unique primers, complementary to the regions flanking the ligation sites of predicted DNA termini and similar to those described previously by Fouts [26], (5′-GGATAAGAATAGATACTATGACC and 5′-TCAACCTGACTAATTCAGCAGC), were used. For z1a, the 5′-CGTACCGTGCTAAACTATCTACA primer was used as the latter primer. The obtained PCR products were sequenced.

Additionally, we performed DNA restriction analysis to visualize termini regions. Digestion patterns were first predicted using a SnapGene Simulate Agarose Gel tool (SnapGene 4.1.9, GSL Biotech LLC, San Diego, CA, USA). Phage DNA was digested with selected restriction enzymes overnight and then either left untreated or heated to 55/85 °C for 10 min, followed by immediate cooling on ice for 15 min. DNA fragments of each sample were separated electrophoretically in a 1% agarose gel. DNA of Lambda phage was used as a positive control for the re-ligation and melting of 5′ *cos* termini. The assembled phage DNA sequences were re-organized according to their identified ends.

### 2.9. Genomic Sequence Analysis and Annotation

Assembled phage sequences were annotated automatically using RAST at its Web site (https://rast.nmpdr.org/, accessed on 15 February 2018) [27]. The annotations were corrected manually based on the results of DNA analysis with the use of BLASTx and NCBI RefSeq database. The closest homologs of proteins encoded by the predicted CDSs were identified using BLASTp, BLASTx, and tBLASTn (https://blast.ncbi.nlm.nih.gov/Blast.cgi, accessed on 20 June 2021). The proteins were queried against Viruses taxid database or *B. cereus* group taxid (when no viral homologs were found). In the case of homologs identified as encoded by bacteria, the PHASTER server was used for verification whether their genes are in prophages (https://phaster.ca/, accessed on 12 January 2021) [28,29]. Additionally, protein motifs were identified with the use of PHMMER tool (https://www.ebi.ac.uk/Tools/hmmer/search/phmmer, accessed on 18 May 2021) [30]. Protein transmembrane domains and signal sequences were predicted with the use of TMHMM 2.0 and SignalP 5.0 at their websites (www.cbs.dtu.dk/services/TMHMM-2.0/ and https://services.healthtech.dtu.dk/service.php?SignalP-5.0, both accessed on 5 November 2021) [31,32]. Selected proteins were additionally analyzed with the use of HHpred (https://toolkit.tuebingen.mpg.de/tools/hhpred, accessed on 16 December 2021) to find their structural homologs [33,34].

BLASTn (https://blast.ncbi.nlm.nih.gov/Blast.cgi, accessed on 20 June 2021) tool was used to find close relatives of tested phages among phages of completely sequenced genomes deposited in GenBank. Percentage identities between the DNA sequences of the tested phages and their close relatives were calculated with the use of Viridic [35]. The phylogenetic relatedness of newly isolated phages with similar phages was determined based on genome-wide sequence similarities calculated by tBLASTx with the use ViPTree (https://www.genome.jp/viptree/, accessed on 23 November 2021) [36], which utilizes the original proteomic tree concept developed by Rohwer and Edwards [37]. In addition, the phylogenetic position of newly isolated phages among closely related phages was determined using BioNumerics v7.6 (Applied Maths, Sint-Martens-Latem, Belgium) by analyzing the major capsid proteins and terminase large subunits. The twelve most similar phage genomes were used as the reference genomes in further comparative studies. The numbers of core proteins within particular phage clades were calculated with the use of CoreGenes 5.0 (https://coregenes.ngrok.io/, accessed on 20 June 2021) with the bidirectional best hit algorithm and E-value 1e-05 [38,39].

Geneious Prime software version 2021.2.1 (Biomatters, Auckland, New Zealand) was used to create a whole-genome synteny map of the newly isolated phages and their close relatives. Nucleotide multiple sequence alignment was performed using MAFFT 7.0 program [40,41]. The corresponding proteins of tested phages and their relatives were analyzed again using BLASTp to determine their amino acid sequence similarity, and color-coding was used to mark the results based on the percentage identity of protein sequences compared. 

### 2.10. Nucleotide Sequences Accession Numbers

GenBank accession numbers: Complete genomic sequences of phages J5a, F16Ba and z1a have been deposited in GenBank, under the accession numbers: MT745955, MT745954, MT745956, respectively.

## 3. Results

### 3.1. Phage Host Range and Morphology

On the basis of the preliminary results of phage sequence analysis, three of the newly isolated phages, vB_BanS-J5a, vB_BanS-F16Ba, and vB_BanS-z1a (J5a, F16Ba, and z1a), were selected for further research.

**Figure 1:**
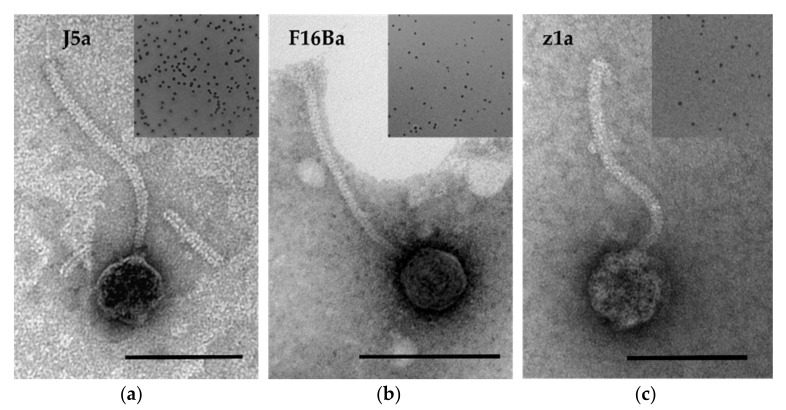
Transmission electron micrographs of (**a**) J5a, (**b**) F16Ba, and (**c**) z1a phage. Plaque morphologies of each phage are shown at the upper right corner of each TEM image. For TEM, the phages were stained with 2% uranyl acetate [42]. The scale bar corresponds to 100 nm.

The host range of the selected phages was investigated with the use of 51 bacterial strains, including four virulent *B. anthracis* strains. Of the tested strains, all three phages could productively lyse only cells of *B. anthracis* strains, as indicated by the formation of single plaques. Notably, *Bacillus* sp. 813+ strains, containing anthrax chromosomal marker gene, turned out to be insensitive to phages. All phages formed plaques of about 1 mm or less in diameter on a layer of each of the sensitive strain cells (Figure 1). Only in the case of phage J5a plaques could one see a faint halo zone at the border of plaques.

Based on the analysis of TEM images, all three phages were taxonomically assigned to the siphovirus morphotype of *Caudoviricetes* class (Figure 1). Their virions are composed of a long, non-contractile tail and a head. The tails of an average length of 188 nm end with a central tail fiber (spike), which is protruding below the baseplate (see Figure 1a). The head diameters (58 nm on average) fit the approximate virion DNA sizes of these phages, estimated based on their migration in PFGE (approximately 40 kb).

### 3.2. Adsorption Efficiency, One-Step Growth Curve, and Optimal MOI

**Figure 2:**
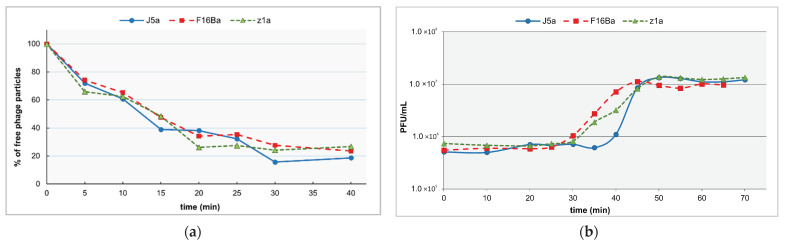
Physiological properties of phages J5a, F16Ba, and z1a: (**a**) adsorption efficiencies; (**b**) one-step growth curves.

The adsorption of all phages to their host cells started quickly upon mixing phages with bacteria, and the adsorption efficiencies were similar (Figure 2a). About 70–85% of phages could be adsorbed upon 30 min of phage addition to bacteria (Figure 2a, Table 1). Results of one-step growth experiments indicated also similar latent periods and burst sizes of all three phages (Figure 2b; Table 1).

Infection of indicator strain cells with each of the phages tested with different MOIs showed that the highest production of phage progeny for all three phages was obtained when phages and bacteria were mixed in the ratio 1:2; thus, MOI 0.5 was used in further experiments.

### 3.3. Sensitivity of Phages to Temperature and pH

**Figure 3:**
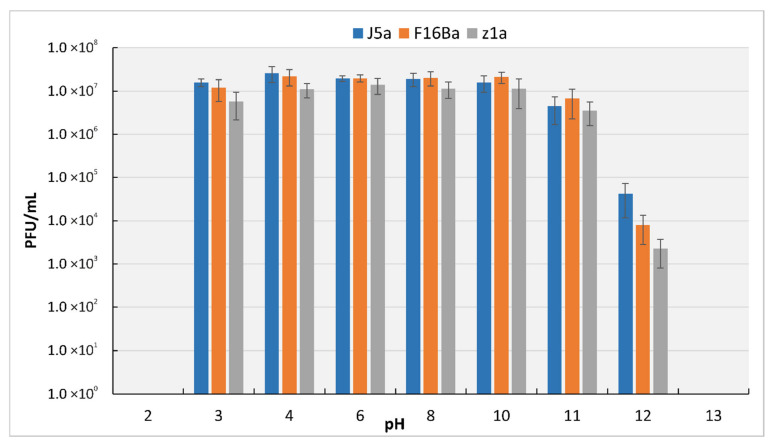
Survivability of phages J5a, F16Ba, and z1a upon incubation at different pHs.

All three phages were stable at pH between 3 and 11, with no statistically significant differences between them. No phage particles remained viable upon treatment in a solution of pH 2 and 13 (Figure 3).

**Figure 4:**
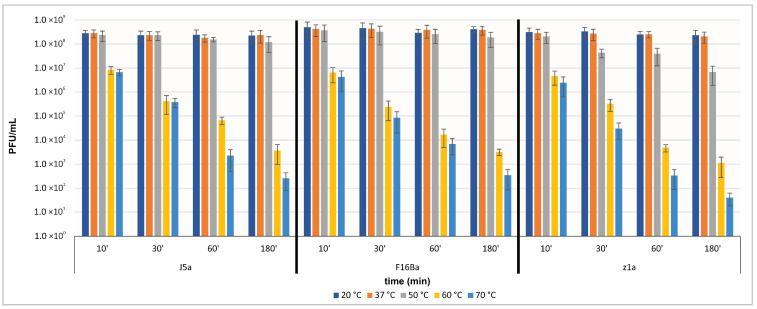
Thermal stability of phages J5a, F16Ba, and z1a.

All three phages appeared to be equally stable at temperatures 20 °C and 37 °C. Their titer was slightly decreasing after prolonged incubation at 50 °C (Figure 4). Phage z1a was the least stable at this temperature, and its titer decreased by about 1.5 orders of magnitude after three hours. At 60 °C and 70 °C, a significant time-dependent decline of phage titers could be observed in the case of all tested phages.

### 3.4. General Features of Phage Genomic Sequences and Identification of Virion DNA Ends

**Figure 5:**
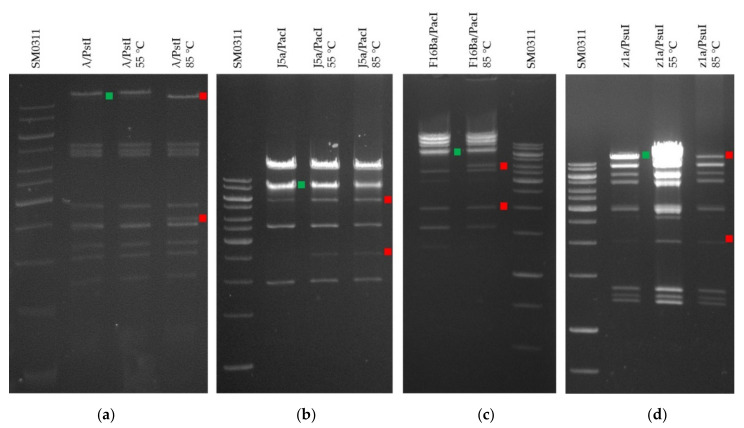
Differences in the migration pattern of denatured and non-denatured restriction fragments of phages J5a, F16Ba, and z1a DNA upon electrophoretic separation in a 1% agarose gel. The fragments that contain *cos* overhangs and migrate separately upon partial denaturation are marked with red squares, while the products of their annealing through the *cos* sequences that are formed under non-denaturing conditions are marked with green squares. (**a**) Phage λ DNA digested with PstI (11,497 + 2560 = 14,057 bp). Differences in bands are visible upon DNA denaturation at 85 °C; (**b**) J5a phage DNA digested with PacI (5448 + 2508 = 7956 bp). Different migration upon denaturation at 85 °C is more clearly visible; (**c**) F16Ba phage DNA digested with PacI after heating at 85 °C (3868 + 1877 = 5745 bp); (**d**) z1a phage DNA digested with PsuI (16,390 + 2203 = 18,413 bp). The DNA ladder used was the SM0311 GeneRuler 1 kb (Thermo Scientific, Waltham, MA, USA). *In silico*-predicted migration patterns are available in Supplementary Data for comparison (Figure S1).

The sequence reads of J5a, z1a, and F16Ba genome library fragments assembled into 40,353, 39,355, and 38,554 bp molecules, respectively. Virion DNA molecules of all three phages appeared to end with 5’ overhangs of CGCCGCCCC sequence as determined with PhageTerm. The presence of *cos* sequences at the ends of virion DNA was consistent with the differences in the electrophoretic migration of mildly denatured and non-denatured restriction-digested phage DNA in a gel (Figure 5). The pattern of bands representing fragments of digested phage DNA changed upon heating, revealing the denatured fragments containing cohesive ends and derived from the fragments annealed through these ends prior to denaturation, like in the case of the Lambda phage (Figure 5).

The summary of general genomic features of phages J5a, F16Ba, and z1a is shown in Table 2. The G+C content in the genomes of all three phages is similar to that of their bacterial host *B. anthracis* (35.1%). The predicted genes can encode 63, 58, and 54 proteins, respectively, and no tRNA.

**Figure 6:**
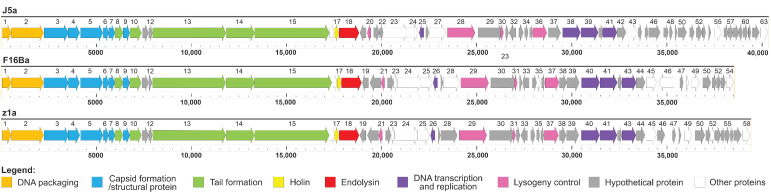
Comparison of schematic genomic maps of phages J5a, F16Ba, and z1a. Genes are color-coded based on their predicted functions.

The new phages are 85.3% (J5a vs. z1a), 81.5% (F16Ba vs. z1a), and 78.0% (F16Ba vs. J5a) identical with each other, and have a similar organization of genes in the genomes (Figure 6). They appear to be closely related to the *B. anthracis* phages: Negev_SA, Carmel_SA, and Tavor_SA. The similarity of new phages with each other and with their closest, previously described relatives is below the demarcation criteria that allow one to classify two phages to the same species (95%) [43], indicating that each of the newly isolated phages represents a new species.

### 3.5. Phylogenetic Analysis

**Figure 7:**
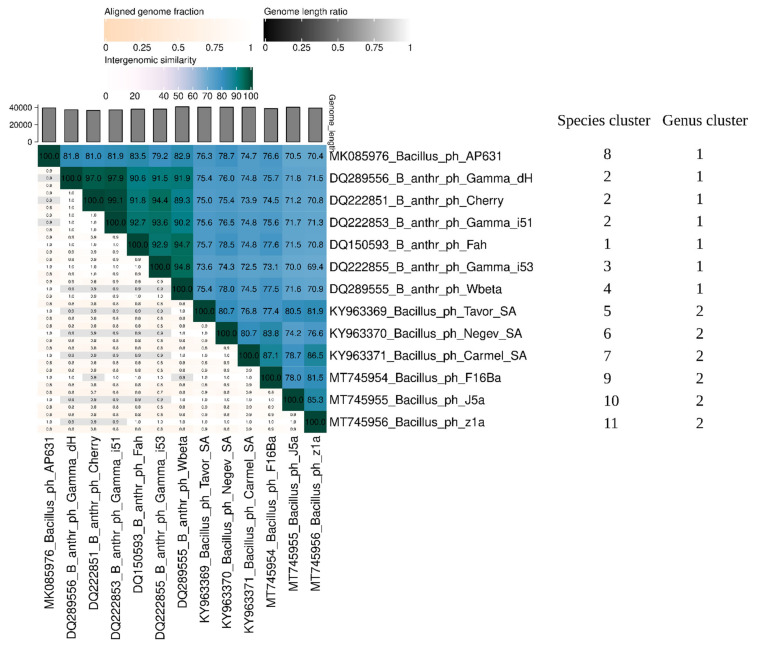
The whole-genome comparison and clustering of phages J5a, F16Ba, z1a, and their closest relatives. The comparison and clustering were performed with the use of Viridic (Virus Intergenomic Distance Calculator; [35]). Different shades of blue in the right half of the heatmap represent different intergenomic similarities (in %) between the genomes of each pair compared, as indicated above the heatmap and specified by numbers. The left half of the heatmap shows three indicator values for each genome pair: aligned fraction of genome 1 for the genome in this row (top value), genome length ratio for the two genomes in this pair (middle value) and aligned fraction of genome 2 for the genome in this column (bottom value). The darker colors represent lower values as indicated above the heatmap.

A search of GenBank for sequences similar to those of J5a, F16Ba, and z1a revealed, in addition to phages Negev_SA, Carmel_SA, and Tavor_SA, seven phages of genomes of over 70% identity, including phage Wbeta, which has been classified to *Wbetavirus* genus of *Caudoviricetes* class, and Wbeta-related phages AP631, Cherry, Fah, Carmel, and three Gamma phage isolates (Figure 7). The genomic sequence of the next closest relative, *Bacillus* phage phIS3501 of *Camtrevirus* genus (GenBank Acc. No. JQ062992), is identical with the sequences of newly isolated phages only in 27–31%. Comparison of J5a, F16Ba, and z1a sequences with the sequences of their closest relatives with the use of Viridic, which computes pairwise intergenomic distances/similarities amongst viral genomes, revealed that all these phages could be classified together with *B. anthracis* phage Wbeta to the *Wbetavirus* genus, based on the phage genus demarcation criteria (70% [43], Figure 7).

**Figure 8:**
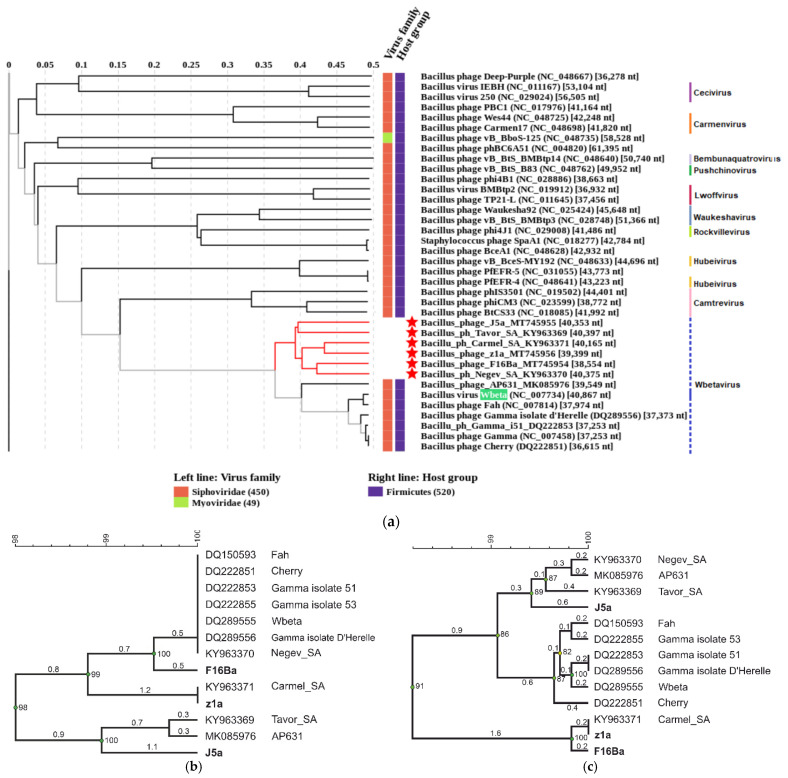
Phylogenetic analysis of J5a, F16Ba, z1a, and the most closely related phages based on whole-genome-wide sequence similarities calculated by tBLASTx with the use of ViPTree [36] (**a**); major capsid protein similarities (**b**); terminase large subunit similarities (**c**). Vertical lines to the right of the whole-genome-based phylogenetic tree in (**a**) mark the phages that have been already classified to particular genera. Dotted vertical lines indicate phages that we propose here to be included in the *Wbetavirus* genus. The name of the prototypical phage of this genus (Wbeta) is highlighted in green. Phages of the proposed J5a clade are indicated with red asterisks. The branches of phylogenetic tree that indicate their separation from the Wbeta clade phages are in red. The phylogenetic trees (**b**,**c**) were clustered using the UPGMA method and verified by the cophenetic correlation coefficient (BioNumerics). Evolutionary distances are shown.

Their clustering, based on the species demarcation criteria, allowed one to divide them into 11 species (Figure 7), grouped into two further clusters that might represent two genera. In support of that, the whole proteome-based phylogenetic tree shows the clustering of these species into two clades (Figure 8a). Phages J5a, F16Ba, and z1a cluster together with phages Negev_SA, Carmel_SA, and Tavor_SA, which were isolated recently in Israel [44], but have not been analyzed in detail (8a). However, as % DNA identities between phages of these two clades in all cases but one exceed 70%, we propose to include all of them in *Wbetavirus* genus together with phage Wbeta of this genus, and to separate them into two clades within this genus, the J5a clade and the Wbeta clade. The distinction of two clades among Wbeta-related phages correlates with differences between the proteomes of phages representing each clade. While all 13 phages share only 31 predicted gene products, the phages of Wbeta clade share 36, and the phages of J5a clade share 41 predicted gene products as calculated with the use of CoreGenes. Phylogenetic trees based on the major capsid protein and large terminase subunit also cluster J5a, F16Ba and z1a separately from historical isolates Wbeta, Gamma, and Cherry (Figure 8b,c).

### 3.6. Comparative Genomic Analysis

The genome sizes of the J5a, F16Ba, and z1a phages and the organization of genes in their genomes resemble those of their relatives from J5a and Wbeta clades. Multiple alignment highlighted the collinearity and similar organization of functional modules in the genomes of the tested and reference phages (Figure S2). Most of the genes are transcribed in one direction (59 out of 63, 48 out of 54, and 53 out of 58, respectively). Searches for the homologs of predicted gene products of J5a, F16Ba, and z1a at the amino acid sequence and structural level and identification of their function-associated amino acid sequence motifs allow one to functionally assign nearly 50% of these phages’ proteins (Tables S2–S4 and S6–S8). Functions of a few more proteins can be predicted only imprecisely, based on the presence in their sequences of putative transmembrane helices or DNA-binding motifs.

The modules at the left ends of the genomes of newly isolated phages and other wbetaviruses encode morphogenetic functions (DNA-packaging, head and tail structure and maturation) and are almost identical (Figure S2; Tables S2–S4). The central and right modules are more diversified. They contain several clade-specific genes and certain genes differentiating particular phages within each clade (Figure S2).

**Figure 9:**
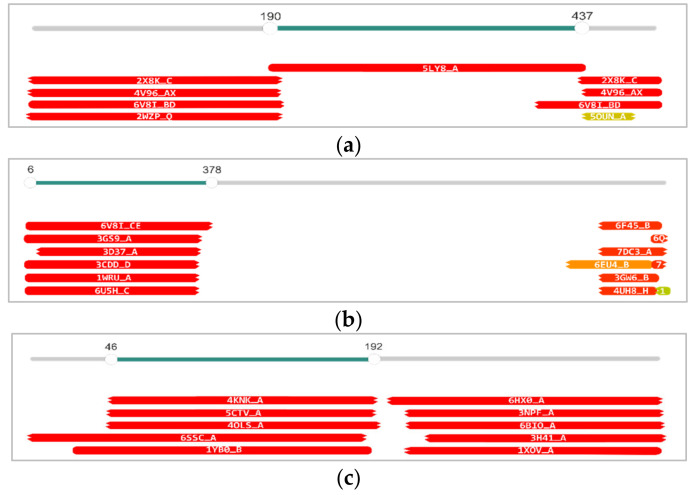
Full sequence HHpred analysis of predicted Dit (J5a_014) (**a**), Tal/RBP (J5a_015) (**b**), and endolysin (J5a_018) (**c**) of J5a bacteriophage. The query sequences are shown as slider bars. The matches to proteins or protein domains from the PDB database are shown as horizontal bars underneath, indicating their coverage with respect to the query. The bars are labeled with the PDB accession numbers of matching structures and color-coded according to their similarity score to the query (from red as the most similar, through orange to green; see the associated data in Table S6).

Similarities between the predicted structures of certain products of the left module genes that encode tail proteins and the structures of tail proteins of some other phages reveal the putative structure of host cell recognition and penetration machinery of wbetaviruses (see Figure 9 and Table S6). The predicted structures of the N- and C-terminal parts of J5a_014 protein and its counterparts in other wbetaviruses are similar to the structures of N- and C-terminal parts of distal tail proteins (Dits) of *Bacillus* phage SPP1, *Lactococcus* phage TP901-1, and *Staphylococcus* virus 80alfa. The central part of these proteins is similar to the *Lactobacillus casei* phage J-1 tail protein carbohydrate-binding module CBM2. Distal tail proteins of the aforementioned phages are localized between the tail tube and tail tip and are proposed to be a docking platform for the tail adsorption apparatus of Gram-positive bacteria siphoviruses, forming a baseplate hub [45,46,47,48]. The central parts of *Lactobacillus casei* phage J-1 Dit and Dit proteins of certain other phages of Gram-positive bacteria bind specific sugar moieties at the surface of bacteria and play a role in the interaction with cells of phage hosts [49,50]. Based on the aforementioned similarities, we conclude that J5a_014 and its counterparts of other wbetaviruses function as Dit proteins of these phages.

The predicted structure of the N-terminal part of J5a_015 and its counterparts in other wbetaviruses is similar to the structure of the N-terminal part of tail-associated lysin (Tal) of *Staphylococcus* virus 80alfa [47] and the N-terminal parts of relevant proteins of certain other phages. The predicted structure of the C-terminal part of J5a_015 is similar to the structure of C-terminal part of the long tail fiber of *Salmonella* phage vB_SenMS16, the tail spike of *Acinetobacter baumannii* phage vB_AbaP_AS12, the L-shaped tail fiber of *Enterobacteria* phage T5, the intramolecular chaperone of endo-*n*-acetylneuraminidase of *Enterobacteria* phage K1F tail spike, and also to the intramolecular autocatalytic protease of *Mus musculus*, a chaperone protein. Intramolecular chaperone domains have been identified at the C-termini of phage long tail fibers or tail spikes of several unrelated phages, including *Enterobacteria* phages K1F and T5. They participate in the formation of a triple-β-helix fold characteristic for these proteins, are autocleaved off the target proteins upon the completion of the folding process, and uncover the receptor-binding domain or exopolysaccharide depolymerase domain [51,52]. Certain proteins similar to Tal proteins at their N-terminal parts, and assigned as Tal/RBPs, contain extensions that function as receptor-binding domains. Based on similarities of different parts of J5a_015 protein to parts of various Tal or Tal/RBP proteins, we conclude that J5a_015 forms a tail spike of wbetaviruses and functions as a Tal/RBP protein.

The central module of wbetaviruses genomes contains genes whose products are involved in host cell lysis, lysogeny control, replication, and transcription.

Holins and endolysins encoded by this module differ significantly between the phages of J5a clade and Wbeta clade (Figure S2; Table S8). The endolysins of J5a clade phages are about 30% larger than those of Wbeta clade phages (~351 aa residues vs. 233 aa residues), and their sequences show weak similarity to those of Wbeta clade phages only in the region encoding the *N*-acetylmuramoyl-l-alanine amidase catalytic domain (27% coverage and 23% identity). Additionally, they have a signal peptide sequence at their N-termini, while endolysins of Wbeta clade phages do not. A search for structural homologs of phage J5a endolysin (J5a_018) with the use of HHpred and a protein-structures database revealed the two-domain structure of this endolysin (Figure 9a). The predicted structure of the region that directly follows the signal peptide sequence (aa 46−192) is highly similar to the predicted structure of *N*-acetylmuramoyl-l-alanine amidase domain (AmiA; 4KNK_A) of bifunctional 1256 aa autolysin AtlA of *Staphylococcus aureus*, to family 2 *N*-acetylmuramoyl-l-alanine amidase (PlyL) of LambdaBa02 prophage of *B. anthracis* (1YB0_B), and to other lysins catalytic domains of similar activity. Residues His-265, His-370, and Asp-384, which directly coordinate the zinc ion in AtlA, as well as the nearby residues Glu-324 and His-382, which participate in catalysis by AtlA [53], are conserved in J5a_018. The predicted structure of J5a_018 C-terminal moiety (aa 199-349), which should represent the cell wall-binding domain (CBD), is highly similar to the structure of CBD of *Listeria* phage A500 endolysin Ply500 (6HX0_A), *Listeria* phage PSA endolysin Ply (1XOV_A), and *B. cereus* γ-d-glutamyl-l-diamino acid endopeptidase YkfC (3H41_A), and to CBD domains of certain other peptidoglycan-cleaving proteins (Figure 9c; Table S6). The CBD domain of Ply500, Ply, and YkfC comprise two copies of beta-barrel SH3b-like repeats [54,55], implying that the CBDs of J5a clade phages endolysins also comprise two copies of beta-barrel SH3b-like repeats.

The genomic regions of J5a clade phages that in the Wbeta clade phages encode 141 aa holins of class III that are nearly identical to the holins of *Bacillus* phages of *Rockvillevirus* and *Camtrevirus* genus (phage phIS3501, GenBank Acc. No. NC_019502, among them) encode two smaller proteins. One of them (XpaF1, 78 aa) contains a predicted transmembrane domain and is similar to hemolysin XhlA 1 family proteins. Certain proteins with XhlA family motif (pfam10779; Tables S2–S4) are cell-surface-associated hemolysins that lyse insect granulocytes and plasmatocytes, and also rabbit and horse erythrocytes [56]. However, proteins similar to XhlA that are encoded by the *Bacillus* phage SPP1 and defective prophage PBSX cell lysis modules, and are 35% and 33%, respectively, identical to XhlA-like proteins of J5a clade phages (as calculated by BLASTp), function as holins [57]. Thus, we assign the holin function also to the XhlA-like proteins of J5a clade phages. The second small protein of the J5a clade lytic module (in J5a: J5a_017, 79 aa) has two transmembrane domains and the C-terminal end rich in lysine residues. These features are characteristic of class II holins [58]. In support of that, this protein is 28% identical to the Hol44 holin of *Oenococcus oeni* phage fOG44 (as calculated by BLASTp), which also has two transmembrane domains and cooperates with a signal peptide-containing endolysin in cell lysis [59,60,61].

The genomic organization of regions immediately downstream of endolysin genes also differs between J5a clade and Wbeta clade phages. In the J5a clade phages, this region contains a gene encoding a predicted membrane protein of unknown function (J5a_019) and, except for J5a, a gene encoding a protein with structural homologies to *E. coli* conjugal transfer protein TrwB. In the Wbeta clade phages, it encodes a predicted lipoprotein.

The next diversified region is immediately downstream of the site-specific recombinase gene (Figure S2). In the prototypical Wbeta phage, it represents the lysogeny control module and contains five genes (wp28–wp32), including CI-like phage repressor, Cro-like repressor, and antirepressor genes [17]. In phage Cherry and those Gamma isolates that are obligatorily lytic relatives of Wbeta, the relevant regions contain large deletions encompassing one or both repressor genes (CI-like and Cro-like) and three preceding genes. None of these deletions is present in the J5a clade phages, suggesting that these phages are able to lysogenize their hosts, at least under certain conditions.

The region preceding the CI-like repressor gene, which in Gamma and Cherry is encompassed by the deletion, in the remaining phages encodes two proteins of the recently identified phage arbitrium communication system, AimR and AimP [62,63]. AimR is the intracellular pheromone receptor, which is responsible for the choice between lysis and lysogeny, dependent on the concentration of a hexapeptide pheromone that is released by phage-infected cells and is the product of processed AimP. Based on protein similarities, AimR/AimP proteins of Wbeta phages are differentiated into two groups, one is represented by most of the J5a clade phages (J5a, z1a, Tavor_SA, and Carmel_SA) while the remaining phages represent the second one. The C-terminal hexapeptides of AimP of these two groups, which correspond to the mature arbitrium system pheromones, differ by one amino acid residue (**T**IKPGG vs. **E**IKPGG). However, the N-terminal amino acid residues of certain arbitrium pheromones were shown to specifically interact with AimR [64]. Moreover, the **E**IKPGG hexapeptide pheromone of Wbeta phage depleted of the initial **E** could not replace the intact pheromone in its action [63]. This may suggest that the arbitrium system of phages J5a, z1a, Tavor_SA, and Negev_SA senses different hexapeptides than does the arbitrium system of other wbetaviruses analyzed in this work.

The most diversified regions of wbetaviruses are upstream of the right ends of their virion DNA (Figure S2). They contain mostly genes of unknown function. In J5a, this region contains eight genes whose predicted products have no homologs among proteins of other Wbeta-related phages (Tables S2–S4). Five hypothetical J5a proteins encoded by this region (J5a_044, J5a_045, J5a_046, J5a_049, and J5a_052), and one hypothetical F16Ba protein (F16Ba_050) have only bacterial homologs. Analysis of bacterial genomes that encode these homologs with PHASTER, which identifies prophage regions in bacterial DNA, revealed that all these proteins are encoded by genes of Wbeta-like prophage regions (Tables S2–S4). The remaining proteins of no homology to gene products of Wbeta-like phages are similar to proteins of unknown functions of other phages.

The rightmost gene of wbetaviruses genomes, which is predicted to encode the HNH endonuclease (HNHE), is conserved. The predicted structure of its product appears to be strikingly similar over its entire length to the structure of the HNHE of deep-sea thermophilic bacteriophage GVE2 of *Geobacillus* sp. Additionally, the amino acid sequences of wbetaviruses HNHEs are 50–51% identical to that of GVE2 HNHE, and the residues shown to be essential for the GVE2 HNHE DNA nicking activity [65] are conserved.

## 4. Discussion

We present in this work the genomic and basic biological characteristics of three newly isolated *B. anthracis* phages—J5a, F16Ba, and z1a. We show that they form together with recently isolated *Bacillus* phages: Carmel_SA, Negev_SA and Tavor_SA a new clade of phages that could be classified to the *Wbetavirus* genus together with historical anthrax phages: Wbeta, three Gamma isolates, Fah, and Cherry (based on the genomic similarity criteria). All these phages are closely related to the prototypical phage of this genus, temperate phage Wbeta. Their virion DNAs end with the 5’ overhangs of identical sequence (CGCCGCCCC) (Section 3.4; [26,66]).

The presence of intact lysis-lysogeny control modules indicates that most of the proposed phages of *Wbetavirus* genus, including J5a, F16Ba, and z1a, are temperate, like the Wbeta phage. The only lytic phages in this genus, Gamma and Cherry, acquired their obligatorily lytic phenotype by the deletion of various regions of this module [17]. The deletion of relevant genomic regions should also allow to obtain the obligatorily lytic derivatives of phages J5a, F16Ba, and z1a.

While the whole-genome comparison of the newly isolated phages to phage Wbeta and other proposed *Wbetavirus* genus representatives indicates the division of these phages into two clades, the pattern of sequence similarities between functionally related products of those genes that differ significantly in sequence in particular phages in several cases is not clade-specific (Figure S2, Tables S5 and S8). For instance, the amino acid sequence differences in the arbitrium system pheromone receptor AimR and its specific pheromone suggest that the specificity of arbitrium system of four phages of J5a clade (J5a, z1a, Carmel_SA, and Tavor_SA) is different than that of the two remaining phages of this clade and all temperate phages of Wbeta clade. The amino acid sequence of site-specific recombinase, as well as the DNA sequences of short regions upstream and downstream of the recombinase genes, is highly similar in three phages of J5a clade (J5a, z1a, and Tavor_SA) but differs from the relevant sequences of the remaining wbetaphages, which are highly similar to each other. Possibly, the recombinases of those two sequence types recognize different attachment sites in the genomes of their hosts.

Significant, clade-specific differences concern the cell lysis genome module of wbetaphages. While the phages of Wbeta clade encode the canonical, 233 aa endolysin and the 141 aa holin of class III (similar to those of the *Rockvillevirus* and *Camtrevirus* phages), the phages of J5a clade encode endolysin of about 351 aa with a signal peptide and two proteins of holin features.

Endolysins of Wbeta clade phages and predicted endolysins of J5a clade phages are *N*-acetylmuramoyl-l-alanine amidases (Tables S2–S4; [20,67]). Results of our analysis indicate that the size difference between these endolysins is associated with the presence of signal peptide in the latter and with the size difference of CBDs of these endolysins (~75 aa vs. 159 aa). Isolated endolysin of Gamma phage (PlyG) can specifically kill cells of *B. anthracis* and other members of the *B. anthracis* cluster [20]. The C-terminal CBD of PlyG binds specifically to the *B. anthracis* cell wall, namely, to secondary cell wall polysaccharides (SCWP) [68,69]. The similarity of the predicted structure of J5a endolysin CBD to structures of those endolysins CBDs that comprise two copies of beta-barrel SH3b-like repeats, such as CBD of Ply500, may suggest that J5a endolysin also binds to a polysaccharide ligand. In the case of Ply500, these SH3-like repeats were shown to be held together by means of swapped beta-strains, which in turn permits the recognition of carbohydrate ligands [54,70]. The elucidation whether similar ligands can be recognized and bound by PlyG and J5a clade endolysins requires further studies.

The presence of signal peptide (SP) in endolysins of J5a clade phages indicates differences in the lysis mediated by these phages and Wbeta clade phages. Certain phages of Gram-positive bacteria encode endolysins that are synthesized in a form of pre-proteins with SP and are transported through a cytoplasmic membrane to reach a cell wall by a SecA-dependent pathway with concomitant removal of SP [60,71]. The lethal functions of holins encoded by these phages have been postulated to fully sensitize bacteria to these endolysins action [72]. The involvement of more than one holin in phage-mediated cell lysis has been also described in the case of certain phages [73,74,75]. It may facilitate the lysis of cells grown in different conditions or cells of different phage hosts [57].

A search for homologs of lytic proteins encoded by the phages of J5a clade indicates a horizontal transfer of lytic module between these phages and other phages infecting strains of various *Bacillus* species. While endolysins of J5a clade phages are highly similar only to each other and to the amidase of *Bacillus paranthracis* strain BC478A prophage phBC6A51 (Acc. No. MCC2432590.1), their more distant orthologs are encoded by certain unclassified *Bacillus* siphophages, *Bacillus cereus* phages of *Cecivirus* genus, and *Bacillus thuringiensis* phages of *Waukeshavirus* genus. It is of notion that in the *Camtrevirus* genus phages, the gene encoding holin of class III corresponding to holins of Wbeta clade phages is also preceded by a gene encoding a protein with the XhlA 1 family motif, like in the phages of J5a clade (see e.g., *Bacillus* phage phIS3501, GenBank Acc. No. NC_019502).

Phages J5a, F16Ba, and z1a exhibited lytic activity exclusively against anthrax strains and not against other strains from the *B. cereus* group that were used in our studies. In contrast, the highly specific flagship *B. anthracis* phage—Gamma—can infect a few *B. cereus* strains in addition to *B. anthracis* [76,77,78]. Therefore, WHO suggests that for identification or detection purposes, the Gamma phage should be used in combination with other tests, and not as a sole means [79]. Phage Fah, which is highly similar to Wbeta and was studied in detail by Minakhin [66], was propagated in a *B. cereus* strain. In the case of other anthrax phages similar to Wbeta: Cherry, Negev_SA, Carmel_SA and Tavor_SA, there is no detailed information on bacterial strains used for their host range analysis.

The first step that determines the infectivity of a given phage for a bacterium is the ability of this phage to recognize and bind to this bacterium. In tailed phages, this interaction involves distal phage tail components. Previous studies resulted in the identification of phage Gamma Gp14 and the corresponding phage Wbeta Wp14 as receptor-binding proteins [17]. Purified Gp14 bound to *B. anthracis* cells and retained this ability when fused to a fluorescent protein [17,80]. Our search of the protein structural database with the use of HHpred and tail proteins of wbetaviruses as queries allowed us to assign Gamma Gp14 and its counterparts in other Wbeta-like phages as distal tail proteins (Dits) and to identify a putative second RBP of these phages, assigned by us as Tal/RBP. A triad of tape-measure protein (TMP), Dit, and Tal (Tal/RBP) is conserved in siphophages’ distal tail parts, although this common scaffold may contain various functional extensions [48]. Additionally, the assignment of Dit and Tal/RBP functions to proteins encoded by the wbetaviruses genes that are directly downstream of the tape-measure protein gene is consistent with the organization of genes encoding the relevant proteins in the genomes of certain other siphoviruses. In the case of wbetaviruses, the organization of these genes appears to be the most similar to that of *Bacillus* phage SPP1, where the Tal/RBP is encoded by a single gene. However, the Dit protein of wbetaviruses is larger than that of SPP1 (496 aa vs. 253 aa) [81]. The difference in the former can be attributed to the presence in its central region of a module similar to the second carbohydrate-binding module (CBM2) of *Lactobacillus* phage J-1 Dit protein (aa 368-614 in J-1 Dit) [49]. The Dit proteins containing the internal CBM domain(s) and assigned as evolved Dits are host recognition and adsorption parts of tails of certain siphophages infecting Gram-positive bacteria, including *Lactobacillus* phages [49], *Lactococcus* phages [49], and *Streptococcus* phages [50,82]. Results of our analysis indicate that they are also tail components of Wbeta-like viruses.

The most distal tail part of phage Wbeta and Wbeta-like phages including our new isolates is a long central tail fiber (spike) (Figure 1a) (see also [17,66]), which is similar to that of *Bacillus* phage TP21-l of *Lwoffvirus TP21* species [83]. It has to be a product of the gene that was previously assigned to encode a minor structural protein (J5a_015 in J5a) [17,66]. Analysis of structural similarities of this protein with the use of HHpred and protein structures database indicated that it corresponds to Tal/RBP proteins of certain other siphoviruses, but has a mosaic structure. Its N-terminal region is similar to that of the phage 80alfa Tal/RBP, the central domain has no significant structural similarity to any proteins of known structure, while the C-terminal domain is similar to the intramolecular chaperons of tail fibers or tail spikes of phages of Gram-negative bacteria. These chaperones have been identified in immature tail fiber and tail spike proteins of evolutionary distant phages [52]. They are required for the correct trimerization and folding of their native proteins and, upon autocatalytic cleavage off, uncover the central receptor-binding or depolymerase domains of these proteins. Siphophages of Gram-positive bacteria that target protein receptors have a straight, central tail fiber directly attached to the tail or to the baseplate [84]. *B. anthracis* protein that was identified as Gamma phage receptor is a sortase-anchored protein, GamR [85]. We propose that the Wbeta-like phage Tal/RBP protein is the one that is responsible for the binding to this receptor, while the Dit protein of these phages binds an unidentified sugar moiety at the surface of *B. anthracis* cells. While phage Gamma Gp14 (Dit) was shown to bind *B. anthracis* and sensitive *B. cereus* cells, whether Gp14 binds to GamR or to other host receptors has not been studied.

We noticed that while the Dit proteins of wbetaviruses, as well as the N-terminal and C-terminal domains of Tal/RBP proteins, are nearly identical, the central domains of Tal/RBPs are highly diversified (Figure S3). Thus, one cannot exclude that a protein receptor is not the same for all these phages. If the specificity of phages J5a, F16Ba, and z1a exclusively to *B. anthracis* strains can be confirmed with the use of a larger collection of *B. cereus* group isolates, including those that can support the propagation of certain Wbeta clade phages, the new phages may appear to be superior over Gamma phage for the detection of *B. anthracis* isolates, when depleted of their lysogeny modules. The specific activity of our phages against *B. anthracis* would be their important advantage [2]. Their tolerance to a wide range of temperatures and pHs and a short latent period, shown in this work, support their possible utility also as anti-*B. anthracis* agents. A preprepared and evaluated phage cocktail that could have bactericidal activity against most *B. anthracis* strains has been suggested as an optimal anti-anthrax agent [86].

## Figures and Tables

**Table 1:** Comparison of adsorption efficiencies, latency times, and burst sizes of phages J5a, F16Ba, and z1a.

Phage	Adsorption (30 min)	Burst Size (PFU/mL)	Latent Period (min)
J5a	84.3%	20	35
F16Ba	72.4%	16.5	25
z1a	75.8%	17	30

**Table 2:** Genomic features of the phages J5a, F16Ba, and z1a.

Phage	Genome Size (bp)	%GC	Number of Predicted Genes	Closest Relative (GenBank Acc. No.)	% Identity
vB_BanS-J5a (J5a)	40,353	35.17	63	*Bacillus* phage Tavor_SA (KY963369.1)	80.5
vB_BanS-z1a (z1a)	39,355	35.10	58	*Bacillus* phage Carmel_SA (KY963371.1)	86.5
vB_BanS-F16Ba (F16Ba)	38,554	34.84	54	*Bacillus* phage Carmel_SA (KY963371.1)	87.1

## Data Availability

The data presented in this study are available on request from the corresponding author.

## Supplementary Materials

The following supporting information can be downloaded at: https://www.mdpi.com/article/10.3390/v14020213/s1, Figure S1: DNA restriction patterns generated in silico by SnapGene for Lambda, J5a, F16Ba, and z1a phages, indicating expected differences between restricted DNA untreated (circular forms) and treated with heat (linear forms), Figure S2: Gene synteny in the genomes of phages J5a, F16Ba, z1a and other known or proposed members of *Wbetavirus* genus, Figure S3: Alignment of amino acid sequences of Tal/RBP proteins (J5a_015 and its counterparts) of J5a, F16Ba, z1a, and other Wbeta-like bacteriophages, Table S1: List of bacterial strains used in this study, Table S2: Bacteriophage J5a genes and their predicted products, Table S3: Bacteriophage F16Ba genes and their predicted products, Table S4: Bacteriophage z1a genes and their predicted products, Table S5: Coordinates of the newly identified or re-annotated CDSs in the genomes of reference phages, Table S6: Results of HHpred analysis of selected J5a phage proteins. Table S7: Results of HHpred analysis of selected F16Ba and z1a phage proteins of no homologs among proteins of J5a phage, Table S8: Amino acid sequence identity analysis between proteins of J5a bacteriophage and relevant proteins or proteins encoded by similar genome regions of other known or proposed members of *Wbetavirus* genus phages.
