# Supplementary material for: Three Novel Bacteriophages, J5a, F16Ba, and z1a, Specific for Bacillus anthracis, Define a New Clade of Historical Wbeta Phage Relatives
Source: Viruses. 2022 Jan 21;14(2):213. doi: 10.3390/v14020213 (PMC8878798; doi:10.3390/v14020213)
Supplement: Supplementary file 1 [file viruses-14-00213-s001.zip › viruses-1551922-supplementary.pdf]

Table S1. List of bacterial strains used in the study.

Species (No.)	Strain	Obtained from	Notes
B. cereus **(11)**	ATCC 10872	MIHE institute collection	
	ATCC 10876	The University of Scranton, Pennsylvania, USA	
	ATCC 11778		
	ATCC 13472	The University of Scranton, Pennsylvania, USA	
	ATCC 14579^{T}	The University of Scranton, Pennsylvania, USA	
	ATCC 19637	The University of Scranton, Pennsylvania, USA	
	ATCC 23261	The University of Scranton, Pennsylvania, USA	
	F16959	The University of Scranton, Pennsylvania, USA	
	F17202	The University of Scranton, Pennsylvania, USA	
	F17289	The University of Scranton, Pennsylvania, USA	
	UW85	The University of Scranton, Pennsylvania, USA	
B. thuringiensis **(11)**	ATCC 33679	MIHE institute collection	
	ATCC 35646	The University of Scranton, Pennsylvania, USA	
	ATCC 10792		
	ATCC 10792^{T}	MIHE institute collection	
	T07-019	The University of Scranton, Pennsylvania, USA	
	T07-128	The University of Scranton, Pennsylvania, USA	
	T07-146		
	T07-151		
	T07-155	The University of Scranton, Pennsylvania, USA	
	T07-202	The University of Scranton, Pennsylvania, USA	
	#35	The University of Scranton, Pennsylvania, USA	
B. anthracis **(5)**	211	Institute of Veterinary Hygiene in Białystok, field office in Łomża	Isolated from spleen of a cow died of anthrax
	34F2	"Antraphyl" vaccine by Phylaxia-Sanofi	Serial no. 021OE2
	1153		Isolated from a died cow
	1583	The University of Scranton, Pennsylvania, USA	Strain 211 clone
	PZH	National Institute of Public Health - National Institute of Hygiene	
B. mycoides **(3)**	ATCC 6462		
	ATCC 21929	The University of Scranton, Pennsylvania, USA	
	K184	The University of Scranton, Pennsylvania, USA	
B. sp. Ba 813+ **(10)**	#6 (I/2)	The University of Scranton, Pennsylvania, USA	
	#7 (II/3)	The University of Scranton, Pennsylvania, USA	
	#12 (S8553/2)	The University of Scranton, Pennsylvania, USA	
	#16 (PJ572)	The University of Scranton, Pennsylvania, USA	
	#17 (094)	The University of Scranton, Pennsylvania, USA	
	#21 (T1197-77)	The University of Scranton, Pennsylvania, USA	
	#28 (3)	The University of Scranton, Pennsylvania, USA	
	#30 (1B)	The University of Scranton, Pennsylvania, USA	
	#31	The University of Scranton, Pennsylvania, USA	
	#3403	The University of Scranton, Pennsylvania, USA	
B. subtilis	ATCC 6633	The University of Scranton, Pennsylvania, USA	

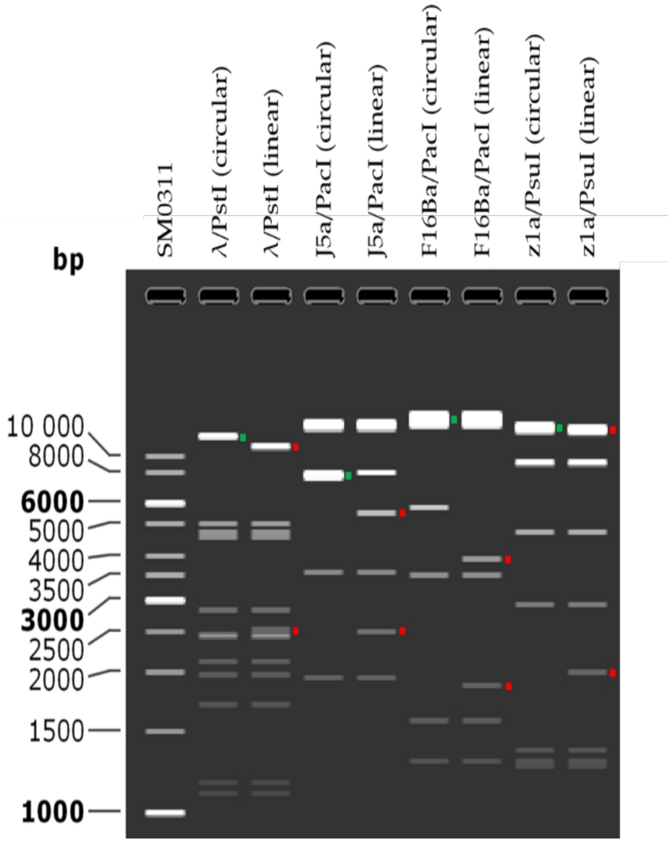

Figure S1. DNA restriction patterns generated *in silico* by SnapGene for Lambda, J5a, F16Ba, and z1a phages, indicating expected differences between restricted DNA untreated (circular forms) and treated with heat (linear forms). DNA ladder used – SM0311 Thermo Scientific GeneRuler 1 kb.

Table S2. Bacteriophage J5a genes and their predicted products. The coverage (in %) and identity (in %) of each protein with its closest homolog from the RefSeq database is as calculated with BLASTp. If no close viral homolog was found bacterial homologs were included. Signal peptides and transmembrane domains are indicated as SP and TMD, respectively.

ORF No.	ORF position (bp)	Length of product (aa)/Predicted molecular mass (kDa)	pI	Predicted function	The closest homolog in GenBank (GenBank acc. no.)	Coverage (%)	Identity (%)	Protein identifier (PHMMER results)
**1.**	63-548	161/18.43	9.92	Terminase small subunit	*B. anthracis* phage Gamma (ABB55435.1)	100	98.14	Terminase_4
**2.**	545-2242	565/65.04	5.03	Terminase large subunit	*B. anthracis* phage Negev_SA (ARW58459.1)	100	99.47	Terminase_1
**3.**	2258-3556	432/48.82	6.23	Portal protein	*B. anthracis* phage Fah (YP_512313.1)	99	97.90	Phage_portal
**4.**	3519-4139	206/23.68	5.11	Prohead protease	*B. anthracis* phage Gamma (ABB55432.1)	100	98.06	Peptidase_S78
**5.**	4178-5356	392/44.42	5.04	Major capsid protein	*B. anthracis* phage AP631 (AZF88350.1)	100	98.21	Phage_capsid
**6.**	5374-5664	96/11.15	4.71	Phage head-tail connector protein	*B. anthracis* phage Carmel_SA (ARW58550.1)	100	98.96	Phage_connect_1
**7.**	5661-5984	107/12.2	6.57	Head-tail adaptor protein	*B. anthracis* phage Gamma (ABA46527.1)	100	98.13	Phage_H_T_join
**8.**	5977-6417	146/16.24	9.62	Hypothetical protein (putative tail-component)	*B. anthracis* phage Negev_SA (ARW58482.1)	100	100	HK97-gp10_like
**9.**	6414-6773	119/13.92	5.44	Conserved phage protein (putative structure protein)	*B. anthracis* phage Gamma (YP_338192.1)	100	98.32	DUF3168
**10.**	6774-7382	202/22.96	5.17	Major tail protein	*B. anthracis* phage Negev_SA (ARW58475.1)	100	96.53	
**11.**	7432-7749	105/11.64	4.46	Hypothetical protein	*B. anthracis* phage Carmel_SA (ARW58548.1)	100	100	
**12.**	7761-7955	64/7.81	5.17	Hypothetical protein	*B. anthracis* phage Carmel_SA (ARW58515.1)	100	98.28	
**13.**	7972-11823	1283/139.32	8.48	Tail length tape-measure protein	*B. anthracis* phage Negev_SA (*ARW58458.1)*	100	97.43	PhageMin_Tail, 3 TMDs

**14.**	11838-13328	496/56.98	6.60	Distal tail protein	*B. anthracis* phage Tavor_SA (ARW58399.1)	100	90.52	Sipho_tail
**15.**	13325-17254	1309/147.41	5.34	Tal/RBP (Tail lysozyme/receptor-binding protein)	*B. anthracis* phage Carmel_SA (ARW58514.1)	100	79.87	Peptidase_S74
**16.**	17293-17529	78/8.67	6.44	XpaF1 protein (hemolysin Xhla1 family protein)	uncultured *Caudoviricetes* phage (ASN69604.1)	100	93.59	XhlaA, TMD
**17.**	17529-17768	79/8.38	9.16	Holin	*B. anthracis* phage Negev_SA (ARW58494.1)	100	93.67	2 TMDs
**18.**	17765-18820	351/39.33	9.20	N-acetylmuramoyl-L-alanine amidase	*B. anthracis* phage Tavor_SA (ARW58406.1)	100	95.16	Amidase_2, SP
**19.**	Complement (18862-19188)	108/12.29	9.70	Hypothetical protein	*B. anthracis* phage Negev_SA (ARW58500.2)	100	92.59	TMD
**20.**	Complement (19244-19438)	64/7.26	9.36	Repressor protein	*B. anthracis* phage Carmel_SA (ARW58551.1)	96	91.94	HTH_3
**21.**	19598-19900	104/12.73	4.81	Hypothetical protein	*Bacillus* phage BVE2 (AUG88604.1)	100	94	
**22.**	19903-20085	60/6.74	9.70	Hypothetical protein	*Bacillus* phage BVE2 (AUG88603.1)	100	90	2 TMDs
**23.**	20095-21384	429/49.69	6.78	FtsK/SpoIIIE family protein (cell division protein FtsK)	*B. anthracis* phage Wbeta (YP_459986.1)	100	95.34	FtsK_SpoIIIE
**24.**	21311-21943	210/25.18	9.84	Conserved phage protein	*B. anthracis* phage Wbeta (YP_459987.1)	100	97.62	Replic_Relax
**25.**	Complement (21977-22213)	78/8.62	6.15	HTH cro/C1-type domain-containing protein (putative transcription regulator)	*B. anthracis* phage Gamma (YP_338207.1)	100	100	HTH_26
**26.**	22377-22496	39/45.97	4.23	Hypothetical protein	*B. anthracis* phage Gamma (YP_338208.1)	100	100	DUF3961, TMD
**27.**	22509-23372	287/33.38	8.85	Conserved phage protein (HTH domain-containing protein)	*B. anthracis* phage Gamma (YP_338209.1)	100	97.56	HTH_36
**28.**	23470-24924	484/57.57	8.96	Site-specific recombinase	*B. anthracis* phage Tavor_SA (ARW58402.1)	88	96.05	Recombinase
**29.**	25083-26369	428/49.89	6.15	Hypothetical protein	*B. anthracis* phage Tavor_SA (ARW58400.1)	100	98.60	

**30.**	26272-26412	46/52.03	8.25	AimP	*B. anthracis* phage Tavor_SA (-)	100	97.83	SP, TMD
**31.**	26518-26673	51/61.08	4.93	Hypothetical protein	*B. anthracis* phage Tavor_SA (ARW58446.1)	100	94.12	
**32.**	Complement (26702-27061)	119/13.75	6.17	Hypothetical protein (HTH cro/C1 family protein)	*B. anthracis* phage Tavor_SA (ARW58421.1)	100	99.16	HTH_3
**33.**	27223-27450	75/8.75	8.53	Hypothetical protein (HTH cro/C1 family protein)	*B. anthracis* phage Tavor_SA (ARW58442.1)	100	100	HTH_3
**34.**	27535-27702	55/6.59	9.15	Hypothetical protein	*B. anthracis* phage Tavor_SA (ARW58453.1)	98	96.30	
**35.**	27776-27931	51/5.99	10.45	Hypothetical protein	*B. anthracis* phage Fah (YP_512340.1)	100	94.12	
**36.**	27949-28695	248/29.14	5.51	Putative antirepressor	*B. anthracis* phage Carmel_SA (ARW58528.1)	100	97.98	ORF6C
**37.**	28762-29415	217/25.81	6.22	Hypothetical protein	*B. anthracis* phage Tavor_SA (ARW58412.1)	100	83.87	
**38.**	29544-30464	306/36.04	8.55	Replication initiation protein	*B. anthracis* phage Negev_SA (ARW58468.1)	100	93.57	Phg_2220_C
**39.**	30480-31391	303/34.94	8.70	Putative DNA replication protein DnaC	*B. anthracis* phage Gamma (YP_338216.1)	100	97.69	
**40.**	31410-31643	77/9.17	7.93	Hypothetical protein	*B. anthracis* phage Gamma (ABA46469.1)	100	93.51	
**41.**	31636-32382	248/28.27	6.16	Sigma-70 family RNA polymerase sigma factor (RNA polymerase sporulation specific sigma factor SigF)	*B. anthracis* phage Negev_SA (ARW58471.1)	100	97.58	Sigma70_r2
**42.**	32379-32855	158/18.97	9.24	Hypothetical protein	*B. anthracis* phage Tavor_SA (ARW58419.1)	100	95.57	
**43.**	32913-33455	180/21.16	5.89	Dimeric dUTPase	*B. anthracis* phage Carmel_SA (ARW58535.1)	100	95.56	dUTPase_2
**44.**	33496-33675	59/6.82	8.03	Hypothetical protein	*B. cereus* AFS001538 (PET50494.1) (Wbeta-like prophage region)	100	98.31	

**45.**	33847-34017	56/6.05	4.50	Hypothetical protein	*B. cereus* FSL W8-0268 (KXY22003.1) (Wbeta-like prophage region)	100	98.21	
**46.**	34047-34649	200/23.67	6.04	Hypothetical protein	*B. thuringiensis* GBAC46 (PRT13233.1) (Wbeta-like prophage region)	100	97	
**47.**	34844-35077	77/9.14	5.25	Hypothetical protein	*B. anthracis* phage Tavor_SA (ARW58441.1)	100	97.40	
**48.**	35098-35259	53/6.12	9.37	Hypothetical protein	*Bacillus* virus IEBH (YP_002154356.1)	100	100	
**49.**	35449-35565	38/43.31	3.96	Hypothetical protein	*B. cereus* VD045 (EJR26235.1)	100	100	
**50.**	35601-36041	146/17.47	4.77	Hypothetical protein	uncultured *Caudoviricetes* phage (ASN69632.1)	48	98.59	
**51.**	36188-36439	83/10.11	5.42	Hypothetical protein	uncultured *Caudoviricetes* phage clone 9AX_2 (ASN69632.1)	98	95.12	
**52.**	36570-36860	96/11.09	10.21	Hypothetical protein	*B. thuringiensis* 4XX2 (MRA88504.1) (Wbeta-like prophage region)	100	96.88	
**53.**	36847-37035	62/7.09	4.61	Hypothetical protein	*B. anthracis* phage Gamma (ABB97503.1)	100	90.32	
**54.**	37188-37310	40/4.88	11.52	Hypothetical protein	*B. anthracis* phage Tavor_SA (ARW58455.1)	100	100	DUF3983
**55.**	37483-37878	131/15.57	6.34	Sigma70_r4 domain-containing protein	*B. anthracis* phage Wbeta (YP_460009.1)	100	98.47	Sigma70_r4
**56.**	37998-38222	74/8.52	5.09	Hypothetical protein	*B. anthracis* phage Gamma (ABA46507.1)	100	86.49	
**57.**	38229-38450	73/8.21	5.61	Hypothetical protein	*B. anthracis* phage AP631 (AZF88397.1)	100	97.26	
**58.**	38456-38785	109/13.4	9.41	Hypothetical protein	*B. anthracis* phage Carmel_SA (ARW58546.1)	100	92.66	
**59.**	38786-38977	63/7.14	4.44	Hypothetical protein	*B. anthracis* phage Tavor_SA (ARW58447.1)	100	100	
**60.**	38997-39251	84/10.18	9.08	Hypothetical protein	*B. anthracis* phage Tavor_SA (ARW58435.1)	100	97.62	

**61.**	39272-39418	48/5.60	8.82	Hypothetical protein	*B. anthracis* phage Gamma (YP_338234.1)	100	100	
**62.**	39450-39863	137/15.33	5.55	Hypothetical protein	*B. anthracis* phage AP631 (AZF88400.1)	89	80.49	
**63.**	39932-40315	127/15.55	9.49	HNH endonuclease	*B. anthracis* phage Gamma (YP_338236.1)	100	98.43	HNH

Table S3. Bacteriophage F16Ba genes and their predicted products. The coverage (in %) and identity (in %) of each protein with its closest homolog from the RefSeq database is as calculated with BLASTp. If no close viral homolog was found bacterial homologs were included. Signal peptides and transmembrane domains are indicated as SP and TMD, respectively.

ORF No.	ORF position (bp)	Length of product (aa)/Predicted molecular mass (kDa)	pI	Predicted function	GenBank phage protein sequences with maximal identity	Coverage (%)	Identity (%)	Protein identifier (PHMMER results)
**1.**	63-548	161/18.47	9.84	Terminase small subunit	*B. anthracis* phage Gamma (ABB55435.1)	100	97.52	Terminase_4
**2.**	545-2242	565/65.17	5.10	Terminase large subunit	*B. anthracis* phage Carmel_SA (ARW58516.1)	100	99.82	Terminase_1
**3.**	2258-3556	432/48.82	6.21	Portal protein	*B. anthracis* phage Fah (YP_512313.1)	99	97.90	Phage_portal
**4.**	3519-4139	206/23.72	5.11	Prohead protease	*B. anthracis* phage Gamma (YP_338187.1)	100	99.51	Peptidase_S78
**5.**	4178-5356	392/44.25	5.03	Major capsid protein	*B. anthracis* phage Gamma (ABB55431.2)	100	99.23	Phage_capsid
**6.**	5374-5664	96/11.17	4.71	Phage head-tail connector protein	*B. anthracis* phage Carmel_SA (ARW58550.1)	100	96.88	Phage_connect_1
**7.**	5661-5984	107/12.19	5.80	Head-tail adaptor protein (phage head-tail joining protein)	*B. anthracis* phage Gamma (ABA46527.1)	100	99.07	Phage_H_T_join
**8.**	5977-6417	146/16.24	9.62	Hypothetical protein (putative tail component)	*B. anthracis* phage Negev_SA (ARW58482.1)	100	100	HK97-gp10_like
**9.**	6414-6773	119/13.95	5.21	Conserved phage protein (putative structure protein)	*B. anthracis* phage Gamma (YP_338192.1)	100	98.32	DUF3168

**10.**	6774-7382	202/22.88	5.16	Major tail protein	*B. anthracis* phage Tavor_SA (ARW58414.1)	100	99.50	
**11.**	7433-7750	105/11.68	4.35	Hypothetical protein	*B. anthracis* phage AP631 (AZF88356.1)	100	100	
**12.**	7780-7956	58/7.02	5.13	Hypothetical protein	*B. anthracis* phage Gamma (YP_338195.1)	100	100	
**13.**	7973-11824	1283/ 139.25	8.35	Tail length tape-measure protein	*B. anthracis* phage Negev_SA (ARW58458.1)	100	99.06	PhageMin_Tail, 3 TMDs
**14.**	11839-13329	496/56.91	7.04	Distal tail protein	*B. anthracis* phage Tavor_SA (ARW58399.1)	100	97.98	Sipho_tail
**15.**	13326-17393	1355/151.99	5.34	Tal/RBP (Tail lysozyme/receptor-binding protein)	*B. anthracis* phage Carmel_SA (ARW58514.1)	100	90.29	Peptidase_S74
**16.**	17432-17668	78/8.70	6.21	XpaF1 protein	uncultured *Caudoviricetes* phage (ASN69604.1)	100	92.31	XhlA, TMD
**17.**	17668-17907	79/8.37	9.16	Holin	*B. anthracis* phage Negev_SA (ARW58494.1)	100	94.94	2 TMDs
**18.**	17904-18959	351/39.41	9.32	N-acetylmuramoyl-L-alanine amidase	*B. anthracis* phage Tavor_SA (ARW58406.1)	100	98.29	Amidase_2, SP
**19.**	Complement (18998-19327)	109/12.36	9.70	Hypothetical protein	*B. anthracis* phage Negev_SA (ARW58500.2)	100	98.17	TMD
**20.**	Complement (19395-19988)	197/22.98	5.26	Hypothetical protein	*B. anthracis* phage Negev_SA (ARW58476.1)	98	96.91	T4SS-DNA_transf
**21.**	Complement (19985-20182)	65/7.39	8.93	Repressor protein	*B. anthracis* phage Carmel_SA (ARW58551.1)	100	93.85	HTH_3
**22.**	20342-20650	102/12.50	5.20	Hypothetical protein	*Bacillus* phage BVE2 (AUG88604.1)	98	89	
**23.**	20647-20829	60/6.69	9.70	Conserved phage protein	*B. anthracis* phage Gamma (ABB72450.1)	100	96.67	2 TMDs
**24.**	20839-22128	429/49.63	6.54	FtsK/SpoIIIE family protein (cell division protein FtsK)	*B. anthracis* phage Wbeta (YP_459986.1)	100	95.57	FtsK_SpoIIIE
**25.**	22055-22687	210/25.20	9.84	Conserved phage protein	*B. anthracis* phage Wbeta (YP_459987.1)	100	98.10	Replic_Relax

**26.**	Complement (22721-22957)	78/8.62	6.15	HTH cro/C1-type domain-containing protein (putative XRE family transcriptional regulator)	*B. anthracis* phage Gamma (YP_338207.1)	100	100	HTH_26
**27.**	23122-23241	39/45.97	4.23	Hypothetical protein	*B. anthracis* phage Gamma (YP_338208.1)	100	100	DUF3961, TMD
**28.**	23254-24117	287/33.37	8.98	Conserved phage protein (HTH domain-containing protein)	*B. anthracis* phage Gamma (YP_338209.1)	100	99.65	HTH_36
**29.**	24193-25638	481/56.42	9.16	Site-specific recombinase	*B. anthracis* phage AP631 (AZF88373.1)	100	99.17	Resolvase
**30.**	25741-27075	444/51.24	7.45	Hypothetical protein	*B. anthracis* phage Negev_SA (ARW58462.1)	100	98.87	
**31.**	26984-27133	49/54.14	9.73	AimP	*B. anthracis* phage Wbeta (-)	100	100	Kinase domain protein (fragment), SP, TMD
**32.**	27272-27394	40/47.03	8.77	Hypothetical protein	*B. anthracis* phage AP631 (AZF88375.1)	100	87.50	
**33.**	Complement (27416-27772)	118/13.62	6.30	Hypothetical protein (HTH Cro/C1 family protein)	*B. anthracis* phage Wbeta (YP_459993.1)	100	99.15	HTH_31
**34.**	27932-28159	75/8.80	8.52	Hypothetical protein (HTH Cro/C1 family protein)	*B. anthracis* phage Wbeta (YP_459994.1)	100	100	HTH_3
**35.**	28199-28357	52/6.06	6.23	Conserved phage protein	*B. anthracis* phage Wbeta (YP_459995.1)	100	100	
**36.**	28429-28584	51/6.04	10.28	Hypothetical protein	*B. anthracis* phage Fah (YP_512340.1)	100	98.04	
**37.**	28602-29348	248/29.17	5.83	Putative antirepressor	*B. anthracis* phage Carmel_SA (ARW58528.1)	100	96.77	ORF6C
**38.**	29376-29753	125/14.80	5.10	Hypothetical protein	*B. anthracis* phage Carmel_SA (ARW58544.1)	100	95.20	
**39.**	29760-30410	216/25.47	7.65	Hypothetical protein	*B. anthracis* phage Carmel_SA (ARW58530.1)	100	92.59	
**40.**	30539-31480	313/36.85	7.74	Replication initiation protein	*B. anthracis* phage Negev_SA (ARW58468.1)	100	96.17	Phg_2220_C

**41.**	31496-32407	303/34.92	8.81	Putative DnaC protein (putative replication protein)	*B. anthracis* phage Wbeta (ABC40434.1)	100	98.68	
**42.**	32426-32659	77/9.14	7.93	Hypothetical protein	*B. anthracis* phage Wbeta (ABC40435.1)	100	98.33	
**43.**	32652-33398	248/28.20	5.72	RNA polymerase sporulation specific sigma factor	*B. anthracis* phage Tavor_SA (ARW58411.1)	100	100	Sigma70_r2
**44.**	33395-33871	158/18.97	9.13	Hypothetical protein	*B. anthracis* phage Tavor_SA (ARW58419.1)	100	99.37	
**45.**	33929-34471	180/21.17	5.26	Dimeric dUTPase	*B. anthracis* phage Carmel_SA (ARW58535.1)	100	97.22	dUTPase_2
**46.**	Complement (34598-35383)	261/31.33	6.79	Beta-galactosidase	*B. anthracis* phage Carmel_SA (ARW58527.1)	100	92.72	Sulfotransfer_2
**47.**	35686-35874	62/7.22	4.54	Hypothetical protein	*B. anthracis* phage Carmel_SA (ARW58563.1)	100	88.71	
**48.**	36027-36149	40/4.81	10.77	Conserved phage protein	*B. anthracis* phage Gamma (ABA46517.1)	100	92.50	DUF3983
**49.**	36322-36717	131/15.60	5.99	Sigma70_r4 domain-containing protein	*B. anthracis* phage Wbeta (YP_460009.1)	100	97.71	Sigma70_r4
**50.**	36902-37321	139/16.25	4.78	Hypothetical protein	*B. thuringiensis* KB1 (KXO02459.1) (Wbeta-like prophage region)	100	94.96	DUF3775
**51.**	37382-37606	74/8.54	6.58	Hypothetical protein	*B. anthracis* phage Carmel_SA (ARW58558.1)	100	94.59	
**52.**	37613-37834	73/8.18	5.86	Hypothetical protein	*B. anthracis* phage Tavor_SA (ARW58445.1)	100	98.63	
**53.**	37841-38095	84/10.24	8.97	Hypothetical protein	*B. anthracis* phage Tavor_SA (ARW58435.1)	100	89.29	
**54.**	38133-38516	127/15.48	9.46	HNH endonuclease	*B. anthracis* phage Fah (YP_512360.1)	100	97.64	HNH

Table S4. Bacteriophage z1a genes and their predicted products. The coverage (in %) and identity (in %) of each protein with its closest homolog from the RefSeq database is as calculated with BLASTp. If no close viral homolog was found bacterial homologs were included. Signal peptides and transmembrane domains are indicated as SP and TMD, respectively.

ORF No.	ORF position (bp)	Length of product (aa)/Predicted molecular mass (kDa)	pI	Predicted function	GenBank phage protein sequences with maximal identity	Coverage (%)	Identity (%)	Protein identifier (PHMMER results)
**1.**	63-548	161/18.52	9.88	Terminase small subunit	*B. anthracis* phage Carmel_SA (ARW58537.1)	100	99.38	Terminase_4
**2.**	545-2242	565/65.17	5.07	Terminase large subunit	*B. anthracis* phage Carmel_SA (ARW58516.1)	100	100	Terminase_1
**3.**	2258-3556	432/48.78	7.08	Portal protein	*B. anthracis* phage Gamma (YP_338186.1)	100	97.69	Phage_portal
**4.**	3519-4139	206/23.7	5.11	Prohead protease	*B. anthracis* phage Negev_SA (ARW58474.1)	100	98.54	Peptidase_S78
**5.**	4178-5356	392/44.22	5.04	Major capsid protein	*B. anthracis* phage Carmel_SA (ARW58522.1)	100	100	Phage_capsid
**6.**	5374-5664	96/11.15	4.71	Phage head-tail connector protein	*B. anthracis* phage Carmel_SA (ARW58550.1)	100	100	Phage_connect_1
**7.**	5661-5984	107/12.28	7.87	Head-tail adaptor protein	*B. anthracis* phage Carmel_SA (ARW58547.1)	100	97.20	Phage_H_T_join
**8.**	5977-6417	146/16.24	9.62	Hypothetical protein (putative tail component)	*B. anthracis* phage Negev_SA (ARW58482.1)	100	100	HK97-gp10_like
**9.**	6414-6773	119/13.94	5.21	Hypothetical protein (putative structure protein)	*B. anthracis* phage Carmel_SA (ARW58545.1)	100	97.48	DUF3168
**10.**	6774-7382	202/22.88	5.27	Major tail protein	*B. anthracis* phage Gamma (YP_338193.1)	100	97.52	
**11.**	7431-7748	105/16.67	4.46	Hypothetical protein	*B. anthracis* phage Carmel_SA (ARW58548.1)	100	99.05	
**12.**	7760-7954	64/7.68	4.97	Hypothetical protein	*B. anthracis* phage Gamma (YP_338195.1)	90	93.10	
**13.**	7971-11822	1283/139.40	7.94	Tail length tape-measure protein	*B. anthracis* phage AP631 (AZF88358.1)	100	91.58	PhageMin_Tail, 3 TMDs

**14.**	11837-13327	496/56.84	7.14	Distal tail protein	*B. anthracis* phage Carmel_SA (ARW58517.1)	100	98.39	Sipho_tail
**15.**	13324-17250	1308/147.40	5.36	Tal/RBP (Tail lysozyme/receptor-binding protein)	*B. anthracis* phage Carmel_SA (ARW58514.1)	100	83.76	
**16.**	17289-17525	78/8.67	6.41	XpaF1 protein	uncultured *Caudoviricetes* phage (ASN69604.1)	100	92.31	XhlA, TMD
**17.**	17525-17764	79/8.32	9.52	Holin	*B. anthracis* phage Tavor_SA (ARW58439.1)	100	100	2 TMDs
**18.**	17761-18816	351/39.23	9.20	N-acetylmuramoyl-L-alanine amidase	*B. anthracis* phage Tavor_SA (ARW58406.1)	100	96.58	Amidase_2, SP
**19.**	Complement (18855-19184)	109/12.39	9.70	Hypothetical protein	*B. anthracis* phage Carmel_SA (ARW58540.1)	100	99.08	TMD
**20.**	Complement (19252-19836)	194/22.74	5.28	Hypothetical protein	*B. anthracis* phage Carmel_SA (ARW58533.1)	100	97.94	T4SS-DNA_transf
**21.**	Complement (19842-20039)	65/7.40	7.87	Repressor protein	*B. anthracis* phage Carmel_SA (ARW58551.1)	100	98.46	HTH_3
**22.**	20199-20507	102/12.48	5.06	Hypothetical protein	*B. anthracis* phage Carmel_SA (ARW58549.1)	100	98.04	
**23.**	20504-20686	60/6.70	9.70	Conserved phage protein	*B. anthracis* phage Gamma (YP_338204.1)	100	96.67	2 TMDs
**24.**	20696-21985	429/49.43	8.80	Cell division protein FtsK	*B. anthracis* phage Carmel_SA (ARW58520.1)	100	98.83	FtsK_SpoIIIE
**25.**	21912-22544	210/25.25	9.84	Conserved phage protein	*B. anthracis* phage Wbeta (YP_459987.1)	100	98.57	Replic_Relax
**26.**	Complement (22577-22813)	78/8.65	5.23	Hypothetical protein (putative phage regulatory protein)	*B. anthracis* phage Carmel_SA (ARW58556.1)	100	100	HTH_26
**27.**	22977-23096	39/4.63	4.56	Hypothetical protein	*B. anthracis* phage AP631 (AZF88371.1)	100	84.62	DUF3961, TMD
**28.**	23112-23975	287/33.50	8.70	Hypothetical protein (HTH domain-containing protein)	*B. anthracis* phage Tavor_SA (ARW58408.1)	100	94.08	HTH_36
**29.**	24073-25527	484/57.53	8.90	Site-specific recombinase	*B. anthracis* phage Tavor_SA (ARW58402.1)	88	97.91	Recombinase

**30.**	25686-26972	428/50.01	6.33	Hypothetical protein	*B. anthracis* phage Tavor_SA (ARW58400.1)	100	98.36	
**31.**	26875-27015	46/50.99	8.25	AimP	*B. anthracis* phage Tavor_SA (-)	100	91.30	SP, TMD
**32.**	27077-27280	67/8.10	6.26	Hypothetical protein	*B. anthracis* phage Tavor_SA (ARW58446.1)	100	94.03	
**33.**	Complement (27308-27667)	119/13.70	6.21	Hypothetical protein (HTH Cro/C1 family protein)	*B. anthracis* phage Tavor_SA (ARW58421.1)	100	100	HTH_3
**34.**	27829-28056	75/8.74	8.55	Hypothetical protein (HTH Cro/C1 family protein)	*B. anthracis* phage Tavor_SA (ARW58442.1)	100	96	HTH_3
**35.**	28143-28307	54/6.46	9.15	Hypothetical protein	*B. anthracis* phage Tavor_SA (ARW58453.1)	100	98.15	
**36.**	28381-28536	51/5.99	10.35	Hypothetical protein	*B. anthracis* phage Fah (YP_512340.1)	100	90.20	
**37.**	28554-29300	248/29.21	5.52	Putative antirepressor	*B. anthracis* phage Carmel_SA (ARW58528.1)	100	97.98	ORF6C
**38.**	29328-29705	125/14.94	4.77	Hypothetical protein	*B. anthracis* phage Carmel_SA (ARW58544.1)	100	99.20	
**39.**	29712-30362	216/25.60	7.65	Hypothetical protein	*B. anthracis* phage Carmel_SA (ARW58530.1)	100	95.83	
**40.**	30491-31459	322/38.04	6.31	Putative phage replisome organizer protein	*B. anthracis* phage Gamma (ABA46496.1)	100	90.37	Phg_2220_C
**41.**	31475-32386	303/34.81	8.91	DNA replication protein DnaC (ATP-binding protein)	*B. anthracis* phage Gamma (YP_338216.1)	100	95.71	
**42.**	32405-32638	77/9.22	5.84	Hypothetical protein	*B. anthracis* phage AP631 (AZF88385.1)	100	89.61	
**43.**	32631-33377	248/28.26	5.90	RNA polymerase sporulation specific sigma [illegible]	*B. anthracis* phage Gamma (YP_338218.1)	100	94.76	Sigma70_r2
**44.**	33374-33850	158/18.86	9.14	Hypothetical protein	*B. anthracis* phage Carmel_SA (ARW58538.1)	100	96.20	
**45.**	33909-34451	180/21.18	5.37	Dimeric dUTPase	*B. anthracis* phage Carmel_SA (ARW58535.1)	100	96.67	dUTPase_2

**46.**	34489-34887	132/14.65	7.66	Hypothetical protein	*Bacillus* phage vB_BthS-HD29phi (QDP43463.1)	100	98.48	SP
**47.**	35281-35469	62/7.18	4.78	Hypothetical protein	*B. anthracis* phage Gamma (YP_338227.1)	100	85.48	
**48.**	35622-35744	40/4.91	11.52	Hypothetical protein	*B. anthracis* phage Tavor_SA (ARW58455.1)	100	97.50	DUF3983
**49.**	35918-36313	131/15.56	6.01	Sigma70_r4 domain-containing protein	*B. anthracis* phage Wbeta (YP_460009.1)	100	98.47	Sigma70_r4
**50.**	36469-36981	170/19.66	5.49	Hypothetical protein	*B. anthracis* phage Carmel_SA (ARW58536.1)	100	98.82	
**51.**	37044-37268	74/8.52	7.93	Hypothetical protein	*B. anthracis* phage Carmel_SA (ARW58558.1)	100	100	
**52.**	37275-37496	73/8.18	5.87	Conserved phage protein	*B. anthracis* phage Gamma (YP_338231.1)	100	95.89	
**53.**	37502-37831	109/13.45	9.41	Hypothetical protein	*B. anthracis* phage Carmel_SA (ARW58546.1)	98	95.33	
**54.**	37832-38023	63/7.14	4.44	Hypothetical protein	*B. anthracis* phage Tavor_SA (ARW58447.1)	100	100	
**55.**	38043-38297	84/10.13	8.96	Hypothetical protein	*B. anthracis* phage Tavor_SA (ARW58435.1)	100	98.81	
**56.**	38294-38464	56/6.49	9.27	Hypothetical protein	*B. anthracis* phage AP631 (AZF88399.1)	100	89.29	
**57.**	38496-38909	137/15.47	5.56	Hypothetical protein	*B. anthracis* phage AP631 (AZF88400.1)	91	79.20	
**58.**	38978-39361	127/15.60	9.53	HNH endonuclease	*B. anthracis* phage Fah (YP_512360.1)	100	96.06	HNH

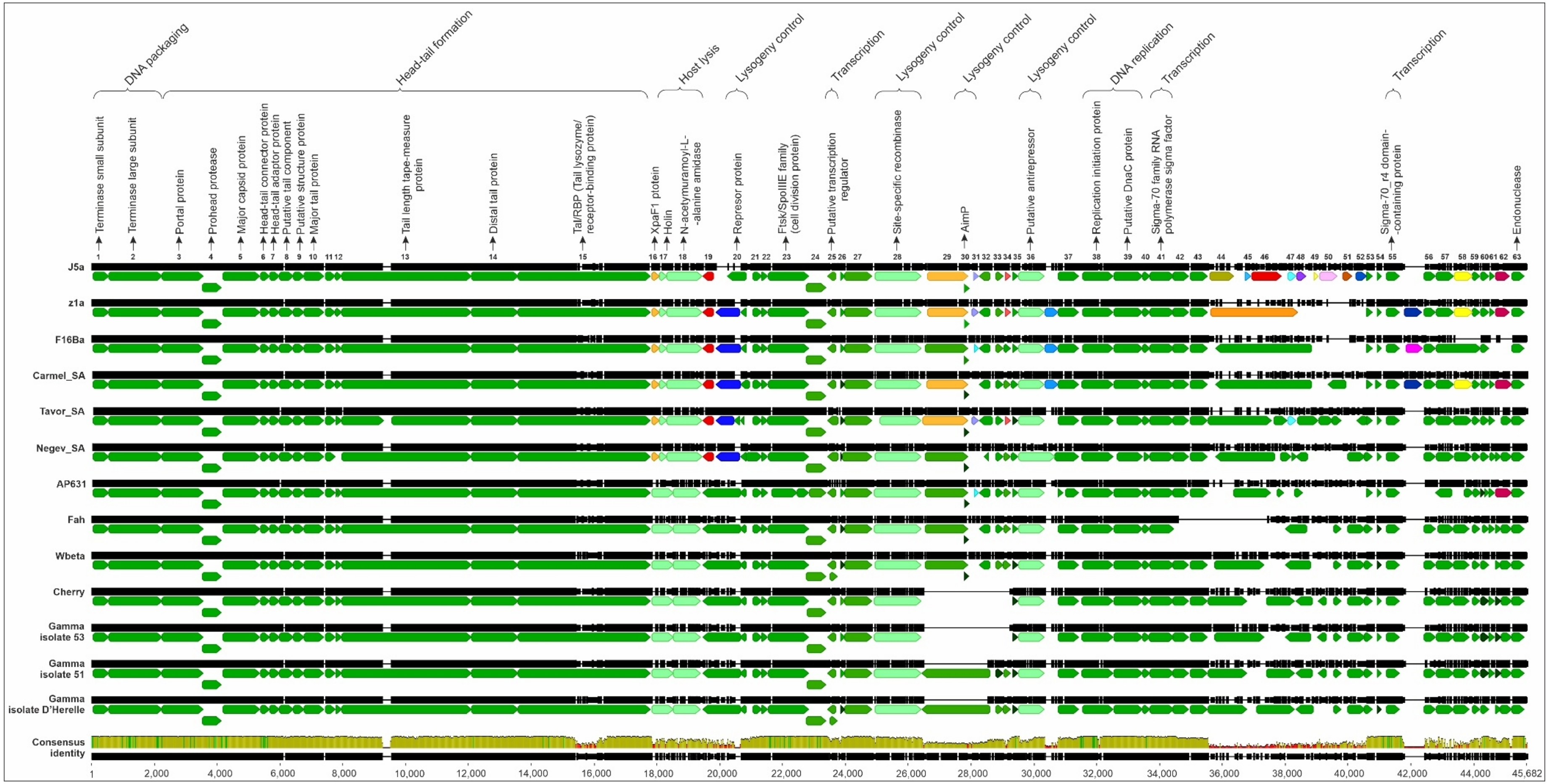

Figure S2. Gene synteny in the genomes of phages J5a, F16Ba, z1a and known or proposed members of *Wbetavirus* genus. The alignment (gapped) was performed with the use of Geneious Prime. Predicted protein-coding genes (CDSs) are color-coded based on the amino acid sequence identity of their products (considered as 70% or higher) or functions. CDSs encoding proteins of J5a, F16Ba and z1a bacteriophages with no homologs among proteins of other wbetaviruses are indicated by unique colors. If two to six phages shared a similar sequence of a protein, the adequate CDSs were given a common color. If more than six phages had a similar sequence of a specific protein, the CDSs were left green. Light green indicates CDSs of similar function but no significant sequence similarity in all genomes. Empty regions in the reference phage genomes were screened revealing the presence of previously unidentified open reading frames. The newly identified genes were re-annotated and were marked with a dark green color. The re-annotated genes are listed in a Table S5 (see also Table S8). Their other genes were also additionally verified what revealed a need to alter some coordinates (these ORFs are marked with an asterisk in a Table S8). The scale length corresponds to the consensus sequence including gaps.

Table S5. Coordinates of the newly identified or re-annotated CDSs in the genomes of reference phages.

Carmel_SA (KY963371.1)	Tavor_SA (KY963369.1)	Negev_SA (KY963370.1)	AP631 (MK085976.1)	Fah (DQ150593.1)	Wbeta (DQ289555.1)	Cherry (DQ222851.1)	Gamma isolate 53 (DQ222855.1)	Gamma isolate 51 (DQ222853.1)	Gamma isolate D'Herelle (DQ289556.1)
23,000-23,119	23,449-23,568	22,999-23,115	26,041-26,163	7,770- 7,946	22,202-22,321	24,711- 24,866	24,710- 24,865	24,852-25,079	22,174-22,293
26,898-27,038	28,849-29,004	26,860-27,009		22,037- 22,156	27,510-27,665	35,273- 35,527	36,734- 36,988	25,349-25,504	25,478- 25,633
	27,347-27,487			34,644- 34,766	37,546-37,668	35,726- 35,917	37,187- 37,378	35,911-36,165	34,052- 34,174
				25,899-26,048	26,064-26,213			36,364-36,555	

Table S6. Results of HHpred analysis of selected J5a phage proteins. The best and most relevant hits are shown, including the coordinates of the matching regions, PDB ID and chain identifier, and the HHpred probability (%) in the matched regions. Only proteins HHPred probability results over 80% are presented. Proteins of J5a phage of no homologs among proteins of F16Ba and z1a phages are marked with an asterisk. Corresponding proteins were assessed as those sharing more than 70% sequence identity, according to BLASTp.

ORF	Protein lenght (aa)	Matched residues (aa)	HHpred best match	PDB ID	Probability(%)
**J5a_014**	496	190-437	Tail component; bacteriophage infection, *Lactobacillus casei*, fluorescence microscopy, Carbohydrate Binding Module, sugar binding protein; 1.28A {*Lactobacillus* phage J-1}	5LY8_A	99.98
		2-196	HYPOTHETICAL PROTEIN 19.1; VIRAL PROTEIN, DISTAL TAIL PROTEIN; 2.95A {BACILLUS PHAGE SPP1}	2X8K_C	99.85
		1-197	Distal tail protein, Receptor-binding protein, Phage baseplate, host adsorption apparatus, genome injection device, VIRAL PROTEIN; 3.8A {*Lactococcus* phage TP901-1}	4V96_AX	99.77
		1-200	Distal Tail Protein, gp58; phage tail, tail tip, tape measure protein, VIRAL PROTEIN; 3.7A {*Staphylococcus* virus 80alpha}	6V8I_BD	99.65
		1-200	LACTOCOCCAL PHAGE P2 ORF15; BASEPLATE, VIRAL PROTEIN; 2.6A {*Lactococcus* Phage P2}	2WZP_Q	98.21
**J5a_015**	1309	6-378	Tail-Associated Lysin, gp59; phage tail, tail tip, tape measure protein, VIRAL PROTEIN; 3.7A {*Staphylococcus* virus 80alpha}	6V8I_CE	99.94
		3-356	Protein gp18; NP_465809.1, prophage tail protein gp18, Structural Genomics, Joint Center for Structural Genomics, JCSG, Protein Structure Initiative; HET: MSE, MLY; 1.7A {*Listeria monocytogenes* EGD-e}	3GS9_A	99.90
		29-355	Tail protein, 43 kDa; tail protein, structural genomics, PSI, MCSG, Protein<br>Structure Initiative, Midwest Center for Structural Genomics, UNKNOWN FUNCTION; 2.1A {*Neisseria meningitidis* MC58} SCOP: b.106.1.1	3D37_A	99.08
		1166-1289	Long tail fiber distal subunit; Bacteriophage, Helical sandwich, Tail fiber, polyglycine, VIRAL PROTEIN; HET: MRD, MPD, IMD; 1.70355818561A {*Salmonella* phage vB_SenMS16}	6F45_B	96.25
		1166-1297	Isoform 2 of Myelin regulatory factor; auto-catalytic protease, protein chaperone. trimeric protein, triple coiled-coil, membrane PROTEIN; HET: MSE; 2.4A {*Mus musculus*}	7DC3_A	95.93
		1166-1283	Endo-N-acetylneuraminidase; Chaperone, Glycosidase, Hydrolase; HET: TAM, PEG; 2.6A {Enterobacteria phage K1F}	3GW6_B	95.76
		1166-1279	L-SHAPED tail fiber protein; viral protein, bacterial viruses, caudovirales, siphoviridae, infection; HET: FLC; 2.52A {ENTEROBACTERIA PHAGE T5}	4UW8_H	95.19
		1100-1271	Tail spike protein; bacteriophage, tailspike, *Acinetobacter baumannii*, gp42, VIRAL PROTEIN; HET: MSE; 1.794A {*Acinetobacter* phage vB_AbaP_AS12}	6EU4_B	87.82
**J5a_018**	351	46-192	Bifunctional autolysin; peptidoglycan, autolysin, amidase, N-acetylmuramoyl-L-alanine amidase, HYDROLASE; HET: IMD, PEG;	4KNK_A	99.76
		45-192	Autolysin; LytA, pneumococci, autolysis, amidase, peptidoglycan complex, antibiotics, hydrolase; HET: NAG, DGL, AMV; 1.05A {*Streptococcus pneumoniae* serotype 4}	5CTV_A	99.54
		45-194	Endolysin; amidase-2 domain, HYDROLASE; HET: ZN; 2.27A {*Staphylococcus* phage GH15}	4OLS_A	99.50
		2-186	N-acetylmuramoyl-L-alanine amidase; amidase, zinc binding, cell wall degradation, endolysine, hydrolase; HET: PO4, GOL; 1.21A {*Clostridium intestinale*}	6SSC_A	99.41
		26-190	prophage LambdaBa02, N-acetylmuramoyl-L-alanine amidase, family 2; N-acetylmuramoyl-L-alanine amidase, PlyL, E.C.3.5.1.28, HYDROLASE; HET: PO4; 1.86A {*Bacillus anthracis*} SCOP: d.118.1.1	1YB0_B	99.38
		199-349	L-alanyl-D-glutamate peptidase; listeria, endolysin, cell wall binding domain, bacteriophage, VIRAL PROTEIN; 1.59A {*Listeria* phage A500}	6HX0_A	98.51
		209-349	putative dipeptidyl-peptidase VI; Structural Genomics, Joint Center for Structural Genomics, JCSG, Protein Structure Initiative, PSI-2, HYDROLASE; HET: GOL, CSA, MSE; 1.72A {*Bacteroides ovatus*}	3NPF_A	97.65
		210-350	Clan CA, family C40, NlpC/P60 superfamily cysteine peptidase; peptidase, NlpC protein, HYDROLASE; 1.2A {*Trichomonas vaginalis*}	6BIO_A	97.61
		220-351	NLP/P60 family protein; NLPC/P60 FAMILY PROTEIN, STRUCTURAL GENOMICS, JOINT CENTER FOR STRUCTURAL	3H41_A	97.35

			GENOMICS, JCSG, PROTEIN STRUCTURE INITIATIVE, PSI-2, HYDROLASE; HET: PG4, MSE, DGL; 1.79A {*Bacillus cereus* ATCC 10987}		
		209-349	Ply protein; alpha/beta hydrolase, multi-domain, hydrolase; 1.8A {*Listeria* phage PSA} SCOP: c.56.5.6, l.1.1.1, b.34.11.4	1XOV_A	96.61
**J5a_019**	108	73-106	*Homo sapiens* Transcription initiation factor IIE, alpha subunit; zinc finger	1VD4_A	98.45
		74-106	*Thermococcus kodakarensis* DNA-directed RNA polymerase subunit P	4QIW_P	98.36
		75-106	*Sulfolobus shibatae* DNA-directed RNA polymerase; transferase, multi-subunit	4AYB_P	98.23
**J5a_024**	210	2-81	*Mycobacterium tuberculosis* (strain ATCC 25618/H37Rv) transcriptional regulator; DNA binding protein	5ZHC_B	97.41
		10-77	*Bacteroides thetaiotaomicron* uncharacterized protein	2L02_A	97.36
		13-80	*Enterobacteria* phage T4 transcription regulatory protein MOTA	1BJA_B	97.33
		128-184	Severe fever with thrombocytopenia virus RNA polymerase	6NTV_A	90.09
		128-184	Toscana virus RNA-dependent RNA polymerase	6QW0_B	89.29
**J5a_027**	287	35-116	*Bacillus subtilis* DNA replication protein DnaD; primosome, DNA-binding protein	2V79_A	98.49
		35-96	*Sulfolobus tokodaii* 109aa long hypothetical transcriptional regulator; helix-turn-helix	2D1H_B	98.22
		35-96	*Geobacillus kaustophilus* HTA426 chromosome replication initiation protein; DnaD	2VN2_C	98.21
**J5a_029**	428	12-384	*Bacillus* phage phi3T AimR transcriptional regulator	5ZVV_B	99.97
		9-386	*Bacillus* phage SPbeta AimR transcriptional regulator; DNA binding protein, peptide binding protein, complex with peptide	5Y24_B	99.97
		7-276	*Bacillus thuringiensis* transcriptional activator PlcR protein	3U3W_B	99.94
**J5a_032**	119	1-70	*Streptococcus suis* 05ZYH33 ComR; *Streptococcus*, Competence, Quorum sensing, ComR, TRANSCRIPTION REGULATOR	5FD4_B	99.40
		1-108	*Streptococcus vestibularis* F0396 transcriptional regulator ComR; RNPP family TPR domain HTH domain bacterial signaling peptide binding, TRANSCRIPTION	6HU8_A	99.28
		2-69	*Staphylococcus aureus* Orf20; SaPI, Repressor, STRUCTURAL PROTEIN	6H49_A	99.20
		1-119	*Clostridium difficile* 630 putative transposon-related DNA-binding protein	3IVP_B	99.18
**J5a_033**	75	4-71	*Cytophaga hutchinsonii* uncharacterized protein	3OMT_B	99.10
		4-72	*Streptococcus suis* 05ZYH33 ComR; *Streptococcus*, Competence, Quorum sensing, ComR, TRANSCRIPTION REGULATOR	5FD4_B	99.07
		1-69	*Bacillus thuringiensis* transcriptional activator PlcR protein	3U3W_B	99.06
**J5a_040**	77	1-63	*Methanococcus maripaludis* S2 conserved uncharacterized archaeal protein	2QZG_C	91.42
		2-63	*Thermoplasma acidophilum* UPF0147 protein Ta0600	2QSB_A	85.94
		12-47	*Drosophila melanogaster* maternal effect protein oskar; 3'-UTR, dimerization, RNA BINDING PROTEIN	5CD8_B	82.23
		1-41	*Bacillus subtilis* PROTEIN (SINI PROTEIN); TRANSCRIPTION REGULATOR	1B0N_B	80.87
**J5a_044***	59	4-55	*Kluyveromyces lactis* (strain ATCC 8585/CBS 2359/DSM 70799/NBRC 1267/NRRL Y-1140/WM37) mRNA decay, decapping, Nudix, nucleotide analog, TRANSLATION	6AM0_D	93.80
		5-58	*Staphylococcus aureus* host factor for Q beta; Hfq, hexamer, RNA binding protein, translational regulator, Sm motif, TRANSLATION	1KQ1_H	91.35
		5-45	*Listeria monocytogenes* protein hfq; LSm/Sm proteins, RNA chaperone, RNA BINDING PROTEIN	4NL2_C	90.52
**J5a_047***	77	10-71	*Pyrococcus furiosus* DNA double-strand break repair rad50 ATPase; zinc finger, rad50, DNA repair, Recombination	1L8D_A	95.80
		12-62	*Saccharomyces cerevisiae* Protein PCF11; zinc-binding, mRNA, RNA processing, RNA binding protein	5M9Z_A	95.59
		10-70	*Pyrococcus furiosus* (strain ATCC 43587/DSM 3638/JCM 8422/Vc1) DNA double-strand break repair Rad50 ATPase; double strand break repair, DNA damage response	6ZFF_B	94.40
**J5a_051***	83	12-39	*Homo sapiens* Myc-binding protein; conserved hypothetical protein	2YY0_D	92.37
		3-44	*Escherichia coli* cell division protein ZAPB	2JEE_C	91.88
		3-44	*Bacillus subtilis* 168 initiation-control protein YabA; YabA, DnaA, DnaN,	5DOL_B	91.42

			Zinc finger, initiation control, replication		
		12-58	*Saccharomyces cerevisiae* (strain ATCC 204508/S288c) mediator of RNA polymerase II transcription subunit 4; transcription initiation	5OQM_h	90.44
**J5a_053**	62	1-30	*Haloarcula marismortui* 50S ribosomal protein LX; 50S ribosomal subunit, ribonucleoprotein, RNA binding, tRNA binding, metal binding	4V9F_6	88.95
		1-30	*Escherichia coli* bacterial RNA polymerase inhibitor	4LLG_N	88.74
		1-30	*Enterobacteria* phage T7 bacterial RNA polymerase inhibitor	2LMC_A	87.03
**J5a_056**	74	2-27	*De Novo* protein coiled-coil Trimer with Glu:Val:Lys Triad	6Q1W_B	88.30
		3-35	Bovine *Adenovirus* 3 assembly intermediate	3ZIF_O	87.40
**J5a_059**	63	35-55	*Clostridium botulinum* Bot.2110.4; mini-protein binder, inhibitor, TOXIN	5VMR_C	93.20
		30-54	*Homo sapiens* coiled-coil domain-containing protein 90B, mitochondrial, general control protein GCN4	6H9M_C	91.48
		12-61	*Silicibacter* sp. uncharacterized peroxidase-related protein; YP_614459.1	2PFX_A	89.37
**J5a_060**	84	2-66	*Homo sapiens* general transcription factor IIE subunit 1	5GPY_A	98.90
		2-70	*Pyrococcus furiosus* transcription factor E	6PLN_A	98.76
		2-71	*Saccharomyces cerevisiae* (strain ATCC 204508/S288c) transcription initiation factor IIE subunit alpha	6GYM_W	98.73
**J5a_061**	48	1-42	*Thermococcus kodakarensis* DNA-directed RNA polymerase subunit P	4QIW_W	97.60
		2-35	*Pyrococcus furiosus* rubrerythrin; Non heme iron peroxidases, oxidative stress	3PWF_B	96.41
		1-46	*Pyrococcus furiosus* hypothetical protein Pf0610; winged-helix like protein with metal binding site	2GMG_A	95.34
**J5a_062**	137	2-45	*Thermococcus kodakarensis* DNA-directed RNA polymerase subunit P	4QIW_W	96.60
		4-45	*Sulfolobus shibatae* DNA-directed RNA polymerase	4AYB_P	95.63
**J5a_063**	127	1-78	HNH endonuclease; Thermophilic bacteriophage, HNH Endonuclease, DNA nicking, HYDROLASE; 1.52A {*Geobacillus* virus E2}	5HOM_A	99.83

Table S7. Results of HHpred analysis of selected F16Ba and z1a phage proteins of no homologs among proteins of J5a phage. The best and most relevant hits are shown, including the coordinates of the matching regions, PDB ID and chain identifier, and the HHpred probability (%) in the matched region. Only proteins HHPred probability results over 80% are presented. Corresponding proteins are given in parentheses. ORFs unique for a particular phage are marked with an asterisk. Corresponding proteins were assessed as those sharing more than 70% sequence identity, according to BLASTp.

ORF	Protein lenght (aa)	Matched residues (aa)	HHpred best match	PDB ID	Probability (%)
**F16Ba_020 (z1a_020)**	197	74-194	*Escherichia coli* conjugal transfer protein trwb; coupling protein, bacterial conjugation, f1-atpase-like quaternary structure, ring helicases	1E9R_A	99.52
		75-196	*Legionella pneumophila* IcmO (DotL); Protein Complex, Secretion, Secretion systems, Gram-negative bacteria, type 4 secretion system, T4SS, coupling protein	6SZ9_A	99.39
		88-197	*Thermoanaerobacter pseudethanolicus* type IV secretory pathway virb4 components-like protein; hydrolase	4AG6_B	99.04
		57-196	*Sulfolobus solfataricus* hera; hydrolase, nura, helicase, translocase	4D2I_B	98.96
**F16Ba_030 ***	444	35-413	*Bacillus* phage SPbeta AimR transcriptional regulator; DNA binding protein, peptide binding protein	5Y24_A	100
		37-410	*Bacillus* phage phi3T AimR transcriptional regulator	5ZVV_A	100
		33-299	*Bacillus thuringiensis* transcriptional activator PlcR protein	3U3W_A	99.92
**z1a_046***	132	1-115	*Streptococcus pneumoniae* lipoprotein; lipocalin, PccL, virulence, transport protein	5CYB_A	97.02
		1-88	*Mycobacterium smegmatis* MC2 51 superoxide dismutase [Cu-Zn]; Respiratory, Supercomplex, SOD, Mycobacterium, ETC, Lipoprotein, ELECTRON TRANSPORT	6ADQ_Z	96.53
		1-118	*Pseudomonas aeruginosa* chaperone CupB2; Ig fold, periplasmic chaperone	3Q48_A	93.31

Table S8. Amino acid sequence identity analysis between proteins of J5a bacteriophage and relevant proteins or proteins encoded by similar genome regions of other known or proposed members of *Wbetavirus* genus phages. Asterisks mark ORFs for which their lengths were altered according to their detailed translation analysis. Green color marks newly added ORFs.

J5a ORF no.	No. of amino acid residues	Known or predicted function	z1a	F16Ba	Carmel_SA	Tavor_SA	Negev_SA	AP631	Fah	Wbeta	Cherry	Gamma isolate 53	Gamma isolate 51	Gamma isolate D'Herelle
**1.**	161	Terminase small subunit	154/161	158/161	153/161	159/161	159/161	158/161	158/161	158/161	158/161	158/161	158/161	158/161
**2.**	565	Terminase large subunit	556/565	555/565	556/565	562/565	562/565	561/565	560/565	559/565	558/565	559/565	558/565	558/565
**3.**	432	Portal protein	424/432	425/432	425*/432	423*/432	422*/432	424/432	424*/432	424/432	424/432	424/432	424/432	424/432
**4.**	206	Prohead protease	202/206	203/206	203*/206	202/206	202/206	203/206	203/206	203/206	203/206	203/206	203/206	203/206
**5.**	392	Major capsid protein	385/392	378/392	379/392	385/392	379/392	385/392	379/392	379/392	379/392	379/392	379/392	379/392
**6.**	96	Head-tail connector protein	95/96	94/96	95/96	95/96	95/96	95/96	95/96	95/96	95/96	95/96	95/96	95/96
**7.**	107	Head-tail adaptor protein	101/107	104/107	100/107	96/107	105/107	104/107	105/107	105/107	105/107	105/107	105/107	105/107
**8.**	146	Hypothetical protein	146/146	146/146	145/146	111/145	146/146	115/145	145/146	145/146	145/146	145/146	145/146	145/146
**9.**	119	DUF3168 domain protein	114/119	117/119	114/119	104/119	117*/119	115/119	118/119	118/119	118/119	118/119	118/119	118/119
**10.**	202	Major tail protein	197/202	194/202	177/202	195/202	195/202	176/202	195/202	195/202	195/202	195/202	195/202	195/202
**11.**	105	Hypothetical protein	104/105	75/105	105/105	77/105	75/105	75/105	75/105	75/105	75/105	75/105	75/105	75/105
**12.**	64	Hypothetical protein	63/64	53/58	57/58	46/58	-	33/37	53/58	53/58	53/58	53/58	53/58	53/58
**13.**	1283	Tail length tape-measure protein	1192/1283	1251/1283	1219/1283	800/845 + 394/438	1250/1283	1219/1283	1203/1283	1203/1283	1203/1283	1203/1283	1203/1283	1203/1283
**14.**	496	Distal tail protein	444/496	447/496	441/496	450/496	448/496	445/496	444/496*	443/496	444/496*	443/496*	444/496*	444/496
**15.**	1309	Tal/RBP (Tail lysozyme/receptor-binding protein)	1155/1308	1083/1355	1075/1321	1076/1332	1036/1332	1095/1309	1024/1288	1057/1331	1023/1288	1025/1288	1025/1288	1057/1331
**16.**	78	XpaF1 protein (hemolysin of Xhla1 protein family)	72/78	72/78	71/78	71/78	71/78	-	-	-	-	-	-	-
**17.**	79	Holin	73/79	78/79	73/79	73/79	74/79	-	-	-	-	-	-	-
		Holin						unrelated (141 aa)	unrelated (141 aa)	unrelated (141 aa)	unrelated (141 aa)	unrelated (141 aa)	unrelated (141 aa)	unrelated (141 aa)
**18.**	351	N-acetylmuramoyl-L-alanine amidase	334/351	331/351	332/351	334/351	333/351	-	-	-	-	-	-	-
		N-acetylmuramoyl-L-alanine amidase						unrelated (233 aa)	unrelated (233 aa)	unrelated (233 aa)	unrelated (233 aa)	unrelated (233 aa)	unrelated (233 aa)	unrelated (233 aa)
**19.**	108	Hypothetical protein	103/109	99/109	102/109*	86/109*	100/109	-	-	-	-	-	-	-
		Predicted lipoprotein						unrelated (163 aa)	unrelated (165 aa)	unrelated (165 aa)	unrelated (163 aa)	unrelated (165 aa)	unrelated (165 aa)	unrelated (165 aa)

			unrelated (194 aa)	unrelated (197 aa)	unrelated (197 aa)	unrelated (163 aa)	unrelated (194 aa)							
		Hypothetical protein, partly similar to J5a ORF033				(60 aa)								
**20.**	64	Repressor protein	58/65	57/65	57/65*	22/67*	51/65	22/70	22/70	22/70	22/70	22/70	22/70	22/70
**21.**	100	Hypothetical protein	88/102	91/102	87/102	86/102	86/102*	93/102	93/102	93/102	93/102	93/102	93/102	93/102
**22.**	60	Hypothetical protein	54/60	55/60	55/60	55/60	54/60	55/60	55/60	55/60	55/60	55/60	55/60	55/60
**23.**	429	Ftsk/SpoIIIE family protein (cell division protein FtsK)	405/429	424/429	407/429	407/429	405/429 *	376/397 + 38/65	409/429	409/429	409/429*	409/429*	409/429*	409/429
**24.**	210	Conserved phage protein	206/210	209/210	204/210*	205/210*	203/210*	204/210*	205/210*	205/210	205/210*	204/210*	205/210*	205/210
**25.**	78	HTH cro/C1-type domain-containing protein (putative transcription regulator)	75/78	78/78	75/78	-	-	-	78/78	78/78	78/78	78/78	78/78	78/78
		Putative transcriptional regulator of Xre family, putative phage repressor				unrelated (72)	unrelated (72)	unrelated (72)						
**26.**	39	Hypothetical protein	29/39	39/39	29/39	26/39	25/39	26/39	39/39	39/39	39/39	39/39	39/39	39/39
**27.**	287	Conserved phage protein (HTH cro/C1-type domain-containing protein)	269/287	281/287	263/287	275/287	269/287	263/287	280/287	280/287	280/287	280/287	280/287	280/287
**28.**	484	Site-specific recombinase	469/484	262/481	260/480	467/484*	263/481	259/481	261/481	261/481	261/481	261/481	261/481	261/481
**29.**	428	Transcriptional regulator (AimR family lysis-lysogeny pheromone receptor)	425/428	215/414	339/428	422/428	217/414	218/414	215/414	215/414	-	-	-	-
**30.**	46	AimP	44/46	14/46	32/46	45/46	14/46	17/46	14/46	14/46	-	-	-	-
**31.**	51	Hypothetical protein	46/51			48/51								
**32.**	119	Hypothetical protein (HTH Cro/C1 family protein), transcriptional regulator of Xr	118/119	90/117	91*/117	118/119	90/117*	91/117*	90/118	90/118	-	-	28/36	28/36
**33.**	75	Hypothetical protein (HTH Cro/C1 family protein)	72/75	61/75	61/75	75/75	61/75	61/75	61/75	61/75	-	-	61/75	61/75
**34.**	55	Hypothetical protein	54/55*	16/52	16/52	53/55*	16/52	15/52	16/52	16/52*	16/52	16/52	16/52	16/52*
**35.**	51	Hypothetical protein	47/51	48/51	43/53	48/51	42/51	48/51	48/51	48/51	48/51	48/51	48/51	48/51
**36.**	248	Putative antirepressor	245/248	242/248	243/248	147/275	23/227	146/275	145/271	145/271	145/271	145/271	145/271	145/271
		Hypothetical protein	unrelated (125)	unrelated (125)	unrelated (125)									
**37.**	217	Hypothetical protein	117/216	121/216	119/216	188/217*	127/216*	166/175 50/55	188/217	188/217	188/217	188/217	188/217	188/217
**38.**	306	Replication initiation protein	283/322	277/313	286/316*	277/323*	291/311*	288/305	276/306*	294/315	294/315*	286/315*	294/315*	294/315

**39.**	303	Putative DNA replication protein DnaC	291/303	298/303	288/303*	299/303*	298/303*	292/303	297/303	297/303	297/303	297/303	297/303	297/303
**40.**	77	Hypothetical protein	72/77	72/77	72/77	72/77	71/77*	72/77	72/77	72/77	72/77	72/77	72/77	72/77
**41.**	248	Sigma-70 family RNA polymerase sigma factor	219/248	241/248	235/248	241/248	242/248	220/248	219/248	219/248	218/248	218/248	219/248	219/248
**42.**	158	Hypothetical protein	148/158	152/158	151/158	152/158	151/158	139/157	-	151/158	151/158	151/158	151/158	151/158
**43.**	180	Dimeric dUTPase	175/180	175/180	172/180	174/180*	170/180	170/180	-	164/180	164/180	164/180	164/180	164/180
**44.**	59	Hypothetical protein, putative RNA-binding protein, structurally similar to Hfq (HHpred 1846677); no phage homologs	-	-	-	-	-	-	-	-	-	-	-	-
		Hypothetical protein, unique for z1a (z1a_045) in *Wbetavirus* genus, but encoded by other *Bacillus* phages (HHpred 6692620), putative lipopotein, contains signal peptide)	132	-	-	-	-	-	-	-	-	-	-	-
		Hypothetical protein, only one similar phage protein with ~40% identity,	-	-	-	91	-	-	-	-	-	-	-	-
		Hypothetical protein, only in Cherry and Gamma, but also in 10 other *Bacillus* phages (HHpred 8770880)	-	-	-	-	-	-	-	-	76	-	72	
		Collagen triple helix repeat-containing protein, similarities to lambdoid phages tail fiber proteins	-	-	-	-	-	351	-	471	-	479	-	-
		Sulfotransferase and mannose-binding protein (HHpred 3635837) unique for F16Ba and Carmel_SA, no homolog in other phages	-	261	261	-	-	-	-	-	-	-	-	-
		Hypothetical protein, unique for AP631 among viruses, possible anti-toxin (HHpred 5513470)	-	-	-	-	-	88	-	-	-	-	-	-
**45.**	56	Hypothetical protein, unique for J5a among viruses, (HHpred 7760117)	-	-	-	-	-	-	-	-	-	-	-	-
**46.**	200	Conserved phage protein, common in other phages but only in J5a among *Wbetavirus*	-	-	-	-	-	-	-	-	-	-	-	-

		phages, present in many strains of *B. thuringiensis* and other bacteria, putative glucose epimerase (HHpred 7740054)												
**47.**	77	Hypothetical protein, only in J5a and Tavor_SA among wbetaviruses; identical or nearly identical proteins are encoded by several *Bacillus* strains	-	-	-	75/77	-	-	-	-	-	-	-	-
**48.**	53	Hypothetical protein, only in J5a among wbetavirus phages, present in 12 other phages; identical or nearly identical protein is encoded by many strains of various *Bacillus* species	-	-	-	-	-	-	-	-	-	-	-	-
**49.**	38	Hypothetical protein, only in J5a among phages	-	-	-	-	-	-	-	-	-	-	-	-
**50.**	146	Hypothetical protein, only in J5a among wbetavirus phages, nearly identical in N-terminal moiety to the N-terminal moiety of an uncultured phage protein 9AX2_46; identical or nearly identical protein in their N-terminal moieties are encoded by many strains of various *Bacillus* species	-	-	-	-	-	-	-	-	-	-	-	-
**51.**	83	Hypothetical protein; only in J5a among wbetavirus phages, nearly identical in N-terminal moiety to the C-terminal moiety of an uncultured phage protein 9AX2_46	-	-	-	-	-	-	-	-	-	-	-	-
**52.**	96	Hypothetical protein; only in J5a among wbetavirus phages, nearly identical to a *B. thuringiensis* protein	-	-	-	-	-	-	-	-	-	-	-	-
**53.**	62	Hypothetical protein; putative inhibitor of RNA polymerase (HHpred 5227104; E=0.025, Probability 96.15)	55/62	57/62	54/62	56/62	56/79	54/62	56/79	56/79	56/79	56/79	56/79	56/79
**54.**	40	Hypothetical protein	39/40	38/40	38/40	40/40	40/40	36/40	39/40	39/40	39/40	39/40	39/40	39/40
**55.**	131	RNA polymerase sigma factor,	127/131	128/131	127/131	127/131	129/131	129/13	129/131*	129/131*	129/131	129/131	129/131	129/131*

		sigma-70 family												
**56.**	74	Hypothetical protein	57/74	56/74	57/74	63/74	64/74	34/88[a]	64/74	64/74	64/74	64/74	64/74	64/74
**57.**	73	Hypothetical protein	65/73	65/73	67/73	65/73	65/73	72/73[a]	65/73*	65/73	65/73	65/73	65/73	65/73
**58.**	109	Hypothetical protein	102/109	-	101/109	-	-	-	-	-	-	-	-	-
**59.**	63	Hypothetical protein	63/63	-	62/63	63/63	63/63	-	62/63	62/63	62/63	62/63	62/63	62/63
**60.**	84	Hypothetical protein, putative transcription initiation factor (HHpred 7442310)	81/84	73/82	79/84	82/84	81/84	75/84*	80/84*	80/84	80/84	80/84	80/84	80/84
**61.**	48	Hypothetical protein; putative DNA-directed RNA polymerase subunit	41/56*	-	41/56*	44/58*	43/73*	40/56*	48/56*	48/56*	48/56*	48/56*	48/56*	48/56*
**62.**	137	Hypothetical protein	132/137	-	98/137	36/88	43/88	99/137	43/88	44/95	43/88	43/88	43/88	44/95
		Hypothetical protein					unrelated (63)		unrelated (63)	unrelated (63)	unrelated (63)	unrelated (63)	unrelated (63)	unrelated (63)
**63.**	127	HNH endonuclease	122/127	124/127	125/127	125/127	123/127	125/127	125/127	125/127	125/127	125/127	125/127	125/127

```
QOQ37199.1   1     MRTPSGILHVVDFKTDQIVAAIQPEDYWDDKRHWELKNNVDMLDFTAFDGTDHAVALQQQNLVLKEVRDGRIVPYVITET  80
QOQ37255.1   1     MRTPSGILHVVDFKTDQIVAAIQPEDYWDDKRHWELKNNVDMLDFTAFDGTDHAVTLQQQNLVLKEVRDGRIVPYVITET  80
ARW58514.1   1     MSTPSGILHVVDFKTDQIVAAIQPEDYWDDKRHWELKNNVEMLDFTAFDGTDHAVTLQQQNLVLKEVRDGRIVPYVITET  80
ARW58396.1   1     MRTPSGILHVVDFKTDQIVAAIQPEDYWDDKRHWELKNNVDMLDFTAFDGTDHAVTLQQQNLVLKEVRDGRIVPYVITET  80
AZF88360.1   1     MRTPSGILHVVDFKTDQIVAAIQPEDYWDDKRHWELKNNVDMLDFTAFDGTDHAVTLQQQNLVLKEVRDGRIVPYVITET  80
YP_459979.1  1     MRTPSGILHVVDFKTDQIVAAIQPEDYWDDKRHWELKNNVDMLDFTAFDGTDHAVTLQQQNLVLKEVRDGRIVPYVITET  80
QOQ37148.1   1     MRTPSGILHVVDFKTDQIIAAIQPQDYWDDKRHWELKNNVDMLDFTAFDGTDDSATLQQQNLVLKEVRDGRIVPYVITET  80
ARW58457.1   1     MRTPSGILHVVDFKTDQIVAAIQPEDYWDDKRHWELKNNVDMLDFTAFDGTDHAVTLQQQNLVLKEVRDGRIVPYVITET  80
ABA46383.1   1     MRTPSGILHVVDFKTDQIVAAIQPEDYWDDKRHWELKNNIDMLDFTAFDGTDHAVTLQQQNLVLKEVRDGRIVPYVITET  80
YP_338198.1  1     MRTPSGILHVVDFKTDQIVAAIQPEDYWDDKRHWELKNNIDMLDFTAFDGTDHAVTLQQQNLVLKEVRDGRIVPYVITET  80
YP_512324.1  1     MRTPSGILHVVDFKTDQIVAAIQPEDYWDDKRHWELKNNIDMLDFTAFDGTDHAVTLQQQNLVLKEVRDGRIVPYVITET  80

QOQ37199.1   81    EKNSDNRSITTYASGAWVQIAKSGVIKPQRIESKTVNEFIDMALIGMKWERGQTDYAGFHTMTIDEFLDPLTFLKKIASL  160
QOQ37255.1   81    EKNSDTRSITTYASGAWIQIAKSGIIKPQRLESKTVNEFIDLALLGMKWQRGITEYAGFHTMTIDEYIDPLTFLKKIASL  160
ARW58514.1   81    EKNSDTRSITTYASGAWIQIAKSGIIKPQRLESKTVNEFIDLALLGMKWQRGITEYAGFHTMTIDEYIDPLTFLKKIASL  160
ARW58396.1   81    EKNSDKRFITTYASGAWIQIAKSGVIKPQRIESKTVNEFMDLALLGMKWQRGITEYAGFHTMTIDEYIDPLTFLKKIASL  160
AZF88360.1   81    EKNSDTRSITTYASGAWIQIAKSGIIKPQRIESKTVNEFIDLALLGMKWQRGVTEYAGFHTMTIDEYIDPLTFLKKIASL  160
YP_459979.1  81    EKNSDTRSITTYASGAWIQIAKSGIIKPQRIESKTVNEFMDLALLGMKWKRGITEYAGFHTMTIDEYIDPLTFLKKIASL  160
QOQ37148.1   81    EKNSDKRSITTYASGAWIQIAKSGIIKPQRIESKTVNEFIDLALLGMKWQRGITEYAGFHTMTIDEYIDPLTFLKKIASL  160
ARW58457.1   81    EKNSDKRSITTYASGAWIQIAKSGVIKPQRIESKTVNEFMDLALLGMKWQRGITEYAGFHTMTIDEYIDPLTFLKKIASL  160
ABA46383.1   81    EKNSDKRSITTYASGAWIQIAKSGVIKPQRIESKTVNEFMDLALLGMKWQRGVTEYAGFHTMTIDEYMDSLTFLKKIASL  160
YP_338198.1  81    EKNSDKRSITTYASGAWIQIAKSGVIKPQRIESKTVNEFMDLALLGMKWQRGVTEYAGFHTMTIDEYMDSLTFLKKIASL  160
YP_512324.1  81    EKNSDKRSITTYASGAWIQIAKSGVIKPQRIESKTVNEFMDLALLGMKWQRGVTEYAGFHTMTIDEYMDSLTFLKKIASL  160

QOQ37199.1   161   FKLEIQYRVEVKGSQIIGWYVDMIQKRGRDTGKEIELGKDLIGVTRIEHTRDICTALVGFVKGEGDKVITIESINKGLPY  240
QOQ37255.1   161   FKLEIRYRVEIKGSRIIGWYVDMIQKRGHDTGKEIELGKDLVGVTRIEHTRNICSALVGFVKGEGDKVITIESINKGLPY  240
ARW58514.1   161   FKLEIRYRVEIKGSRIIGWYVDMIQKRGHDTGKEIELGKDLVGVTRIEHTRNICSALVGFVKGEGDKVITIESINKGLPY  240
ARW58396.1   161   FKLEIRYRVEIKGSRIIGWYVDMIQKRGHDTGKEIELGKDLVGVTRIEHTRNICTALVGFVKGEGDKVITIESINKGLPY  240
AZF88360.1   161   FKLEIRYRVEIKGSRIIGWYVDMIQKRGHDTGKEIELGKDLVGVTRIEHTRNICTALVGFVKGEGDKVITIESINKGLPY  240
YP_459979.1  161   FKLEIRYRVEIKGSRIIGWYVDMIQKRGHDTGKEIELGKDLVGVTRIEHTRNICSALVGFVKGEGDKVITIESINKGLPY  240
QOQ37148.1   161   FKLEIRYRVEVKGSKIIGWYVDMIQKRGYDTGKEIELGKDLVGVTRIEHTRNICTALVGFVKGEGDKIITIESINNGLPY  240
ARW58457.1   161   FKLEIRYRVEIKGSRIIGWYVDMIQKRGHDTGKEIELGKDLVGITRIEHTRNICTALVGFVKGEGDKVITIESINKGLPY  240
ABA46383.1   161   FKLEIRYRVEIKGSKIIGWYVDMIQKRGHDTGXEIELGKDXVGVTRIEHTRNICTALVGFVKGEGDKVITIESINKGLPY  240
YP_338198.1  161   FKLEIRYRVEIKGSKIIGWYVDMIQKRGHDTGKEIELGKDLVGVTRIEHTRNICTALVGFVKGEGDKVITIESINKGLPY  240
YP_512324.1  161   FKLEIRYRVEIKGSKIIGWYVDMIQKRGHDTGKEIELGKDLVGVTRIEHTRNICTALVGFVKGEGDKVITIESINKGLPY  240

QOQ37199.1   241   IVDADAFQRWNEHGQHKFGFYTPETEELDMTPKRLLTLMEIELKKRVNSSISYEVEAQSIGRIFGLEHELINEGDTIKIK  320
QOQ37255.1   241   IVDADAFQRWNEHGQHKFGFYTPETEELDMTPKRLLTLMEIELKKRVNSSISYEVEAQSIGRIFGLEHELINEGDTIKIK  320
ARW58514.1   241   IVDADAFQRWNEHGQHKFGFYTPETEELDMTPKRLLTLMEIELKKRVNSSISYEVEAQSIGRIFGLEHELINEGDTIKIK  320
ARW58396.1   241   IVDADAFQRWNEHGQHKFGFYTPETEELDMTPKRLLTLMEIELKKRVNSSISYEVEAQSIGRIFGLEHELINEGDTIKIK  320
AZF88360.1   241   IVDADAFQRWNEHGQHKFGFYTPETEELDMTPKRLLTLMEIELKKRVNSSISYEVEAQSIGRIFGLEHELINEGDTIKIK  320
YP_459979.1  241   IVDADAFQRWNEHGQHKFGFYTPETEELDMTPKRLLTLMEIELKKRVNSSISYEVEAQSIGRIFGLEHELINEGDTIKIK  320
QOQ37148.1   241   IVDADAFQRWNEHGQHKFGFYTPETEELDMTPKRLLTLMEIELKKRVNSSISYEVEAQSIGRIFGLEHELINEGDTIKIK  320
ARW58457.1   241   IVDADAFQRWNEHGQHKFGFYTPETEELDMTPKRLLTLMEIELKKRVNSSISYEVEAQSIGRIFDLEHELINEGDTIKIK  320
ABA46383.1   241   IVDADAFQRWNEHGQHKFGFYTPETEELDMTPKRLLTLMEIELKKRVNSSISYEVEAQSIGRIFGLEHELINEGDTIKIK  320
YP_338198.1  241   IVDADAFQRWNEHGQHKFGFYTPETEELDMTPKRLLTLMEIELKKRVNSSISYEVEAQSIGRIFGLEHELINEGDTIKIK  320
YP_512324.1  241   IVDADAFQRWNEHGQHKFGFYTPETEELDMTPKRLLTLMEIELKKRVNSSISYEVEAQSIGRIFGLEHELINEGDTIKIK  320

QOQ37199.1   321   DTGFTPALYLEARVIAGDESFTDPTQDKYEFGDYREIVNQNEELRKIYNRILSSLGNKQEMIDQLDKLVQEANETASNAK  400
QOQ37255.1   321   DTGFTPELYLEARVIAGDESFTDPTQDKYEFGDYREIVNQNEELRKIYNRILSSLGNKQEMIDQLDKLVNEANETASNAK  400
ARW58514.1   321   DTGFTPELYLEARVIAGDESFTDPTQDKYEFGDYREIVNQNEELRKIYNRILSSLGNKQEMIDQLDKLVNEANETASNAK  400
ARW58396.1   321   DTGFTPELYLEARVIAGDESFTDPTQDKYEFGDYREIVNQNEELRKIYNRILSSLGNKQEMIDQLDKLVNEANETASNAK  400
AZF88360.1   321   DTGFTPELYLEARVIAGDESFTDPTQDKYEFGDYREIVNQNEELRKIYNRILSSLGNKQEMIDQLDKLVQDANETASNAK  400
YP_459979.1  321   DTGFTPELYLEARVIAGDESFTDSTQDKYEFGDYREIVNQNEELRKIYNRILSSLGNKQEMIDQLDRLVQEANETASNAK  400
QOQ37148.1   321   DTGFTPELYLEARVIAGDESFTDPTQDKYEFGDYREIVNQNEELRKIYNRILSSLGNKQEMIDQLDKLVQDANETASNAK  400
ARW58457.1   321   DTGFTPALYLEARVIAGDESFTDPTQDKYEFGDYREIVNQNEELRKIYNRILSSLGNKQEMIDQLDKLVNEANETASNAK  400
ABA46383.1   321   DTGFTPELYLEARVIAGDESFTDPTQDKYEFGDYREIVNQNEELRKIYNRILSSLGNKQEMIDQLDKLVNEANETASNAK  400
YP_338198.1  321   DTGFTPELYLEARVIAGDESFTDPTQDKYEFGDYREIVNQNEELRKIYNRILSSLGNKQEMIDQLDKLVNEANETASNAK  400
YP_512324.1  321   DTGFTPELYLEARVIAGDESFTDPTQDKYEFGDYREIVNQNEELRKIYNRILSSLGNKQEMIDQLDKLVNEANETASNAK  400

QOQ37199.1   401   KESEAAKALAEKVQENIKNNTVDIIEAMNPPTTGLKPFKTLWRDISNGKPGILKIWTGKAWESVVPDVESVKKETLDQVN  480
QOQ37255.1   401   KESEAAKALAEKVQENIKNNTVEIIEAKNPPTTGLKPFKTLWRDISNGKPGILKIWTGTAWESVVPDVESVKEETLDQVN  480
ARW58514.1   401   KESEAAKTLAEKVQENIKNNTVEIIESKNPPTTGLKPFKTLWRDISNGKPGILKIWTGTAWESVVPDVESVKKETLDQVN  480
ARW58396.1   401   KESEAAKALAEKVQENIKNNTVEIIESKNPPTTGLKPFKTLWRDISNGKPGILKIWTGIAWESVVPDVESVKKETLDQVN  480
AZF88360.1   401   KESEAAKTLAEKVQENIKNNTVEIIESKNPPTTGLKPFKTLWRDISNGKPGILKIWTGTAWESVVPDVESVKKETLDQVN  480
YP_459979.1  401   KESEAAKTLAEKVQENIKNNTVEIIESKNPPTTGLKPFKTLWRDISIGKPGILKIWTGTAWESVVPDVESVKKETLDQVN  480
QOQ37148.1   401   KESEAAKALAEKVQENIKNNTVEIIESKNPPSTGLKPYKTLWRDISSGKPGILKIWTGTAWESVVPDVESVKKETLDQVN  480
ARW58457.1   401   KESEAAKTLAEKVQENIKNNTVEIIESKNPPTTGLKPFKTLWRDIGNGKPGILKIWTGTAWESVVPDVESVKKETLDQVN  480
ABA46383.1   401   KESEAAKALAEKVQENIKNNTVEIIESKNPPTTGLKPFKTLWRDISNGKPGILKIWTGTAWESVVPDVESVKKETLDQVN  480
YP_338198.1  401   KESEAAKALAEKVQENIKNNTVEIIESKNPPTTGLKPFKTLWRDISNGKPGILKIWTGTAWESVVPDVESVKKETLDQVN  480
YP_512324.1  401   KESEAAKALAEKVQENIKNNTVEIIESKNPPTTGLKPFKTLWRDISNGKPGILKIWTGTAWESVVPDVESVKKETLDQVN  480

QOQ37199.1   481   KDIESTKTELNQKVQEAQKQATGQFNEVQEGLQGVSRTISNIENKQGEIDKKVTKFEQDSSGFKTSIETLTKKDTEISSK  560
QOQ37255.1   481   KDIETTKTELNQKVQEAQNQATGQFNEVKESLQGVSRTIFDVQNEQGNINKKVTQIEQTSDGFKTSIESLTKNDTEISGK  560
ARW58514.1   481   KDIETTKTELNQKVQEAQNQATGQFNKVQEGLQGVSRTISNIENKQGEIDNKITKFEQDSSGFKTSIESLTKKDTEISNK  560
ARW58396.1   481   KDIETTKTELNQKVQEAQNQATGQFNKVQEGLQGVSRTISNIENKQGEIDKKVTKFEQDSSGFKTSIESLTKKDTEISNK  560
AZF88360.1   481   KDIETTKTELNQKVQEAQNQATGQFNEVKESLQGVSRTISDVQNEQGNINKKVTQIEQTSDGFKTSIETLTKKDNEISNK  560
YP_459979.1  481   KDIATTKTELNQKVQEAQNQATGQFNEVKESLQGVSRTISNVENKQGEIDKKITKFEQDSSGFKTSIESLTKKDTEISNK  560
QOQ37148.1   481   KDIEITKTELNQKVQEAQNQATGQFNEVQEGLQGVSRTISSIENKQGEIDKKVTKFEQDSNGFKTSIESLTKKDGEISNK  560
ARW58457.1   481   KDIETTKTELNQKVQEAQNQATGQFNEVQEGLQGVSRTISNIENKQGEIDKKVTKFEQDSSGFKTSIESLTKKDTEISNK  560
ABA46383.1   481   KDIESTKTELNQKVQEAQNQATGQFNEVKESLQGVSRTITNVENKQGEIDKKVTKFEQDSSGFKQSIEELTKKDGQITEK  560
YP_338198.1  481   KDIESTKTELNQKVQEAQNQATGQFNEVKESLQGVSRTITNVENKQGEIDKKVTKFEQDSSGFKQSIEELTKKDGQITEK  560
YP_512324.1  481   KDIESTKTELNQKVQEAQNQATGQFNEVKESLQGVSRTITNVENKQGEIDKKVTKFEQDSSGFKQSIEELTKKDGQITEK  560

QOQ37199.1   561   LNTVELTVEGTKKTISDVQQTTNDLKKTTTDIKEEAGKISTKLEQVEART----VGGENWLINTGP--------------  622
QOQ37255.1   561   LNTVESTVEGTKKTISDVQQTTNDLKKTTTEIKEEAGKISTKLEQVEART----VGGENWLINTGR--------------  622
ARW58514.1   561   LNTVESTVEGTKQTISDIQSDTESLRKTTNEIKTTAEGTKETLTQLETKVNDIKIGGPNLFINSGF--------------  626
ARW58396.1   561   LNTVESTVEGTKKTISDIQSDTTSLKQTTTEIKEQAGKISEKLTSVEQKYDNMKIGGQNFYKQKSF--------gaagGT  632
AZF88360.1   561   LNTVESTVEGTKKTISDIQSDTTSLKQTTTEIKEQAGRISEKLTSVEQKYDNMKIGGQNFYKQKSF--------gaagGT  632
YP_459979.1  561   LNTVESTVEGTKKTISEVQQTTNDLKKKTTEIEEKAGKITEKLTSLETREVNVR----NYVINSDFsnvtnswigitnAT  636
QOQ37148.1   561   LNTVELTVEGTKKTISDVQQTASELTKTTTEIKEEAGRTKERMEQINSKVDGLEVGATNLIDGTEF--------inanGW  632
```

```
ARW58457.1    561   LNTVESTVEGTKKTISDIQSDTTSLKQTTTEIKEQAGKISEKLTSVEQKYDNMKIGGQNFYKQKSF--------gaagGT  632
ABA46383.1    561   VNTIESTVEGTKKTISDVQQTTSDLKKTTTEIKEEAGKISEKLMSVETKVNSDKAGGRNLLL----------------GS  624
YP_338198.1   561   VNTIESTVEGTKKTISDVQQTTSDLKKTTTEIKEEAGKISEKLMSVETKVNSDKAGGRNLLL----------------GS  624
YP_512324.1   561   VNTIESTVEGTKKTISDVQQTTSDLKKTTTEIKEEAGKISEKLMSVETKVNSDKAGGRNLLL----------------GS  624

QOQ37199.1    623   ----------NEKPQTIG-------------------------------MVGGAQVNKAKF---------AVQ-------  645
QOQ37255.1    623   ----------NQRPQTIG-------------------------------MSGGAQVNKAGQ---------SFN-------  645
ARW58514.1    627   ----------QNELFSWY-------------------------------FHNGKSAIIVSE---------ADE-------  649
ARW58396.1    633   -------TVKYDENNKWWditipvga--------------s-gswkgILYNNKNAKLLVGRAYTISYEIYADE-------  683
AZF88360.1    633   -------TVKYDENNKWWditipvga--------------s-gswkgILYNNKNAKLLVGRAYTISYEIYAEE-------  683
YP_459979.1   637   -------LFKFVDVNISEasaikkgl--------------qitsnkaFVYQKLPADVFKKKKGIASCYINVSS-------  688
QOQ37148.1    633   arwgsygTISVIKESSLPslptpgslvietkvngaeqavpn-gtqvgMRSSDRKFKVKKGQKYTVSFNVATSElgwlldy  711
ARW58457.1    633   -------TVKYDENNKWWditipvga--------------s-gswkgILYNNKNAKLLVGRAYTISYEIYADE-------  683
ABA46383.1    625   -------NVKYEKTDYL------------------------------INQYSLTENFFAGEEYTFVIKGSVPQ-------  660
YP_338198.1   625   -------NVKYEKTDYL------------------------------INQYSLTENFFAGEEYTFVIKGSVPQ-------  660
YP_512324.1   625   -------NVKYEKTDYL------------------------------INQYSLTENFFAGEEYTFVIKGSVPQ-------  660

QOQ37199.1    646   PgEYIVLecSDH----TDSFYQ---FHLDNTKMGDFEKSKDMTLSLDLQNDvhvdFIlfqfinGVWSENVQKGVPASN-V  717
QOQ37255.1    646   E-DYMIVecTDH----TDSFYQ---FHLDNTKMGDYEKGKDMTFSIDLQNDvpidLIvfqfinGVWTENLYSRFPVDN--  715
ARW58514.1    650   K-VIKFIpkDGE----SGGVYQRVGAKGNSTVTVPFEKEQDYVVTVWLKSSndnqKL------KISAEGLKSQFVNVGKV  718
ARW58396.1    684   V-IPTAI--DIN----NFGVATSTGTNDNDVVAKRIMRTPKTIAGQWVKVS----AT------FIMPDNITQDFYDNSVI  746
AZF88360.1    684   V-IPTAI--DIN----NFGVATSTGTNDNDVVAKRIMRTPKTIAGQWVKVS----AT------FIMPDNITQDFYDNSVI  746
YP_459979.1   689   F-TP-GT--DYP----RLYMRFTYDQNGTEKQYYAILKQQEVTNG-WIRIS--------------IPFDTTGYTGELKEV  745
QOQ37148.1    712   I-YIMYT--DNA----NQRIPT-INTLDFPIIAKINDRENNN----YYRVK----FT------F----TATKDD-DNAYL  764
ARW58457.1    684   V-IPTAI--DIN----NFGVATSTGTNDNDVVAKRIMRTPKTIAGQWVKVS----AT------FIMPDNITQDFYDNSVI  746
ABA46383.1    661   G-QKFGI--WQNggssNVGYATSVYANGITYVT---FKAVATTSGNERKLS-----L------YNYPSNTTKAIVE----  719
YP_338198.1   661   G-QKFGI--WQNggssNVGYATSVYANGITYVT---FKAVATTSGNERKLS-----L------YNYPSNTTKAIVE----  719
YP_512324.1   661   G-QKFGI--WQNggssNVGYATSVYANGITYVT---FKAVATTSGNERKLS-----L------YNYPSNTTKAIVE----  719

QOQ37199.1    718   wkrevwtfnidTRATGWglRLRFARN------TNsigkrfrFKKTKLEKGSIPT-------------DFSKSTYELEQSV  778
QOQ37255.1    716   wsrrsftfkidSRATGWglRLRFERN------ENskgkkyrFKKAKLEKGSVPT-------------DFSKSTYELEQSV  776
ARW58514.1    719   wkkhefkfkgtGVGNG---TITFYAD------VSpigteyfLKKPKVEMGNIATEYTTALEEQVTTDEFTKKTTEIEKSV  789
ARW58396.1    747   -----------GVGNGW--TPTKITN------IK-------IRNMQLEEGNIPTSYRIPSEDQVTTDEFTKKTTEIEKSV  800
AZF88360.1    747   -----------GVGNGW--TPTKITN------IK-------IRNMQLEEGNIPTSYRIPSEDQVTTDEFTKKTTEIEKSV  800
YP_459979.1   746   -----------RVNIAT--ADTTTID------AT-------FTGIMVTFGDLIESWNLAPEDGVTQGVFQSKTTEIEKSV  799
QOQ37148.1    765   -----------LIG-GN--TKRALTGsngyawIR-------VNALKVEKGTIATDWDASNNDKVSLPVFEQKTTDIEKSV  823
ARW58457.1    747   -----------GVGNGW--TPTKITN------IK-------IRNMQLEEGNIPTSYRIPSEDQVTTDEFTKKTNEIEKSV  800
ABA46383.1    720   ----------------W---------------------------VALYKGNKPQDWTPAPEEQVTTDEFTKKTIEITKSV  756
YP_338198.1   720   ----------------W---------------------------VALYKGNKPQDWTPAPEEQVTTDEFTKKTIEITKSV  756
YP_512324.1   720   ----------------W---------------------------VALYKGNKPQDWTPAPEEQVTTDEFTKKTIEITKSV  756

QOQ37199.1    779   DGVKTTVTNVQNSQAGFEKRMSTVEQTASGLSSTVSNLNNVVSDQGKKLTEANTKLEQQATAINAKVELKQVEDYVAGFK  858
QOQ37255.1    777   DGVKTTVSKVQDSQVGFEKRVTTVEQTASGLSTTVSSLNNVVSDQGKKLTEANTKLEQQATAIGAKVELKQVEDYVAGFK  856
ARW58514.1    790   DGVKTTVTNVQNSQAGYEKRMSNVEQTASGLSSTVSNLNNVVSDQGKKLTEANTKLEQQATAIGAKVELKQVEDYVAGFK  869
ARW58396.1    801   DGVKTTVTNVQNSQAGFEKRMSNVEQTATGLSSTVSNLNNVVSDQGKKLTEANTKLEQQATAIGAKVELKQVEDYVAGFK  880
AZF88360.1    801   DGVKTTVTNVQNSQAGFEKRMSNVEQTATGLSSTVSNLNNVVSDQGKKLTEANTKLEQQATAIGAKVELKQVEDYVAGFK  880
YP_459979.1   800   DGVKTTVTNVQNSQAGFEKRMSNVEQTATGLSSTVSNLNNVVSDQGKKLTEANTKLEQQATAIGAKVELKQVEDYVAGFK  879
QOQ37148.1    824   EGIKTTVTKVQDSQAGFEKRMSTVEQTASGLSSTVSNLNNVVSDQGKKLTEANTKIEQQATAIGAKVELKQVEDYVAGFK  903
ARW58457.1    801   DGIKETITKVENNQSGFENRVATVEKDATSIKQNVSLIQNTQTEQGKQLQEAKAGWENTAKALEGKVELKQVEDYVAGFK  880
ABA46383.1    757   DGIKETITKVENNQSGFDKRVATVEKDATAIKQNVSLIQNTQTEQGRQLQEAKAGWENTAKALEGKVELKQVEDYVAGFK  836
YP_338198.1   757   DGIKETITKVENNQSGFDKRVATVEKDATAIKQNVSLIQNTQTEQGRQLQEAKAGWENTAKALEGKVELKQVEDYVAGFK  836
YP_512324.1   757   DGIKETITKVENNQSGFDKRVATVEKDATAIKQNVSLIQNTQTEQGRQLQEAKAGWENTAKALEGKVELKQVEDYVAGFK  836

QOQ37199.1    859   IPELKQTVNQNKQDLLNELANKLATEQFNQKMTLIDNRFIINEEGLNAAAKKTEVYTKTQADGQFAKDSYVRDMESRLQL  938
QOQ37255.1    857   IPELKQTVNQNKQDLLNELANKLATEQFNQKMTLIDNRFTINEQGINAAAKKTEVYTKTQADGQFAADSYVRDMESRLQI  936
ARW58514.1    870   IPELKQTVDKNKQDLLGELANKLATEQFNQKMTLIDNRFTINEQGINAAAKKTEVYTKTQADGQFATGSYVRDMESRLQL  949
ARW58396.1    881   IPELKQTVNQNKQDLLDELANKLATEQFNQKMTLIDNRFIINEQGINAAAKKTEVYTKTQADGQFAKDSYVRDMESRLQL  960
AZF88360.1    881   IPELKQTVNQNKQDLLDELANKLATEQFNQKMTMIDNRFIINEQGINAAAKKTEVYTRIQADGQFAKDSYVRDMESRLQL  960
YP_459979.1   880   IPELKQTVDKNKQDLLDELANKLATEQFNQKMTLIDNRFTINEQGINAAAKKTEVYTKTQADGQFATDSYVRDMESRLQL  959
QOQ37148.1    904   IPELKQTVNQNKQDLLDELANKLATEQFNQKMTLIDNRFTINELGINAAAKKTEVYTIEQANGQFAKDSYVRDMETRLQL  983
ARW58457.1    881   IPELKQTVNQNKQDLLDELANKLATEQFNQKMTMIDNRFTINEQGINAAAKKTEVYTKTQADGQFATGSYVRDMETRLQL  960
ABA46383.1    837   IPELKQTVNQNKQDLLDELANKLATEQFNQKMTLIDNRFTINEQGINAAAKKTEVYTKTQADGQFATGSYVRDMETRLQL  916
YP_338198.1   837   IPELKQTVNQNKQDLLDELANKLATEQFNQKMTLIDNRFTINEQGINAAAKKTEVYTKTQADGQFATGSYVRDMETRLQL  916
YP_512324.1   837   IPELKQTVNQNKQDLLDELANKLATEQFNQKMTLIDNRFTINEQGINAAAKKTEVYTKTQADGQFATGSYVRDMETRLQL  916

QOQ37199.1    939   TEKGVSISVKENDVIAAINMSKENIKLNAARIDLVGKVNAEWIKAGLLSGCQIRTSNTDNYVSLDDQFIRLYERGVARAF  1018
QOQ37255.1    937   TEKGVSISVKENDVIAAFNMSKENIKLNAARIDLVGKVNAEWIKAGMLSGCQIRTSNTDNYVSLDDQFIRLYEKGVARSF  1016
ARW58514.1    950   TEKGVSISVKENDVIAAINMSKENIKLHAARIDLVGKVNAEWIKAGILSGCQIRTSNTDNYVSLDDQFIRLYEKGVARSF  1029
ARW58396.1    961   TEKGVSISVKENDVIAAINMSKENIKLNAARIDLVGKVNAEWIKAGLLSGCQIRTSNTDNYVSLDDQFIRLYERGVARAF  1040
AZF88360.1    961   TEKGVSISVKENDVIAAINMSKENIKLNAARIDLVGKVNAEWIKAGLLSGCQIRTSNTDNYVSLDDQFIRLYERGVARAF  1040
YP_459979.1   960   TEKGVSISVKENDVIAAINMSKENIKLNAARIDLVGKVNAEWIKAGLLSGCQIRTSNTDNYVSLDDQFIRLYERGVARAF  1039
QOQ37148.1    984   TEKGVSISVKENDVIAAINMSKENIKLNAARIDLVGKVNAEWIKAGLLSGCQIRTSNTDNYVSLDDQFIRLYERGVARAF  1063
ARW58457.1    961   TEKGVSISVKENDVIAAINMSKENIKLNAARIDLVGKVNAEWIKAGLLSGCQIRTSNTDNYVSLDDQFIRLYERGVARAF  1040
ABA46383.1    917   TEKGVSISVKENDVIAAINMSKENIKLNAARIDLVGKVNAEWIKAGLLSGCQIRTSNTDNYVSLDDQFIRLYERGVARAF  996
YP_338198.1   917   TEKGVSISVKENDVIAAINMSKENIKLNAARIDLVGKVNAEWIKAGLLSGCQIRTSNTDNYVSLDDQFIRLYERGVARAF  996
YP_512324.1   917   TEKGVSISVKENDVIAAINMSKENIKLNAARIDLVGKVNAEWIKAGLLSGCQIRTSNTDNYVSLDDQFIRLYERGVARAF  996

QOQ37199.1    1019  LGHYRRSDGAVQPTFILGSDEKTNAPEGTLFMSQAGAGWPGAYASIGISDGIVDGAVQKSVYWELQRNGISVLNANDYQA  1098
QOQ37255.1    1017  LGHYRRTDGSVQPTFILGTDEKTSAPAGALFMSQAGAGWSGAYASIGISDGIVDGTVQKSVYWELQRNGLSVLNANDYHV  1096
ARW58514.1    1030  LGHYRRTDGSVQPTFILGTDEKTSAPAGALFMSQAGAGWSGAYASIGISDGIVDGTVQKSVYWELQRNGLSVLNANDYHV  1109
ARW58396.1    1041  LGHYRRSDGAVQPTFILGSDEKTNAPEGTLFMSQAGAGWSGAYASIGISNGIVDGAVQKSVYWELQRNGLSVLNANDYHV  1120
AZF88360.1    1041  LGHYRRTDGAVQPTFILGSDEKTNAPEGTLFMSQAGAGWPGAYASIGISNGIVDGAVQKSVYWELQRNGLSVLNANDYHV  1120
YP_459979.1   1040  LGHYRRSDGAVQPTFILGSDEKTNAPEGTLFMSQAGAGWSGAYASIGISNGIVDGAVQKSVYWELQRNGLSVLNANDYHV  1119
QOQ37148.1    1064  LGHYRRSDGAVQPTFILGSDEKTNAPEGTLFMSQAGAGWSGAYASIGISNGIVDGAVQKSVYWELQRNGLSVLNANDYHV  1143
ARW58457.1    1041  LGHYRRSDGAVQPTFILGSDEKTNAPEGTLFMSQAGAGWSGAYASIGISNGIVDGAVQKSVYWELQRNGLSVLNANDYHV  1120
ABA46383.1    997   LGHYRRSDGAVQPTFILGSDEKTNAPEGTLFMSQAGAGWSGAYASIGISNGIVDGAVQKSVYWELQRNGLSVLNANDYHV  1076
YP_338198.1   997   LGHYRRSDGAVQPTFILGSDEKTNAPEGTLFMSQAGAGWSGAYASIGISNGIVDGAVQKSVYWELQRNGLSVLNANDYHV  1076
YP_512324.1   997   LGHYRRSDGAVQPTFILGSDEKTNAPEGTLFMSQAGAGWSGAYASIGISNGIVDGTVQKSVYWELQRNGLSVLNANDYHV  1076

QOQ37199.1    1099  FYTGNGNWYFRRGKPGLYQTSLVLEDNGSDADLRLPNVTLRNSRVAGYTGVIQMKSSVTQNGWGAIQGNFMSPSLREYKS  1178
QOQ37255.1    1097  FYAGNGSWYFRRGKPGLYQTSLVVEDNSTDSDLRLPNITLRNSRAAGYTGIIQVKSPVTQNGWGSVQGNFMSPSLREYKS  1176
```

```
ARW58514.1   1110  FYAGNGSWYFRRGKVGLYQTSLVVEDNGTDSDLRLPNITLRNSRAAGYTGIIQVKSPVTQNGWGSVQGNFMSPSLREYKS  1189
ARW58396.1   1121  FYAGNGSWYFRRGKPGLYQTSLVVEDNSTDSDLRLPNVTIRNSRAAGYTGVIQLKSPVTQNGWGAVQGNFMTPSLREYKS  1200
AZF88360.1   1121  FYAGSGSWYFRRGKPGLYQTSLVVEDNSTDSDLRLPNVTIRNSRAAGYTGVIQLKSPVTQNGWGAVQGNFMTPSLREYKS  1200
YP_459979.1  1120  FYAGNGNWYFRRGKPGLYQTSLVVEDNSTDSDLRLPNVTIRNSRAAGYTGVIQLKSPVTQNGWGAVQGNFMTPSLREYKS  1199
QOQ37148.1   1144  FYAGNGSWYFRRGKTGLYQTSLVLEDNGTDSDLRLPNVTIRNSRAAGYTGVIQLKSSVTQNGWGAVQGNFMTPSLREYKS  1223
ARW58457.1   1121  FYAGNGSWYFRRGKPGLYQTSLVVEDNSTDSDLRLPNVTIRNSRAAGYTGVIQLKSPVTQNGWGAVQGNFMTPSLREYKS  1200
ABA46383.1   1077  FYAGNGNWYFRRGKPGLYQTSLVVEDNSTDSDLRLPNVTIRNSRAAGYTGVIQLKSPVTQNGWGAVQGNFMTPSLREYKS  1156
YP_338198.1  1077  FYAGNGNWYFRRGKPGLYQTSLVVEDNSTDSDLRLPNVTIRNSRAAGYTGVIQLKSPVTQNGWGAVQGNFMTPSLREYKS  1156
YP_512324.1  1077  FYAGNGNWYFRRGKPGLYQTSLVVEDNSTDSDLRLPNVTIRNSRAAGYTGVIQLKSPVTQNGWGAVQGNFMTPSLREYKS  1156

QOQ37199.1   1179  NIRDVSFSALEKIRNLKIRQFNYKNAVNELYQMREEKDPNDPPLTTQDIKTYYGVIVDEADEDFIDESGKGIHLYSYASI  1258
QOQ37255.1   1177  NIRDVSFSALEKIRNVRVREFNYKNAVNELYKMREEKDPNDPPLTIQDIKTYYGAIVDESDEAFIDESGKGIHLYSYASL  1256
ARW58514.1   1190  NIRDVSFSALEKIRNVRVREFNYKNAVNELYKMREEKDPNDPPLTIQDIKTYYGAIVDESDEAFIDESGKGIHLYSYASL  1269
ARW58396.1   1201  NIRDIPFSALEKIRSLKIRQFNYKNAVNELYRMREEKSPNDPPLTTEDIKTYYGLIVDECDEMFVDESGKGIHLYSYASI  1280
AZF88360.1   1201  NIRDIPFSALEKIRSLKIRQFNYKNAVNELYKMREEKSPNDPPLTIEDIKTYYGLIVDECDEMFVDESGKGIHLYSYASI  1280
YP_459979.1  1200  NIRDISFSALEKIRSLKIRQFNYKNAVNELYRMREEKSPNDPPLTTEDIKTYYGLIVDECDEMFVDESGKGIHLYSYASI  1279
QOQ37148.1   1224  NIRDISFSALEKIRSLKIRQFNYKNAVNELYRMREERSPNAPPLTTEDIKTYYGLIVDECDEMFVDESGKGIHLYSYASI  1303
ARW58457.1   1201  NIRDIPFSALEKIRSLKIRQFNYKNAVNELYRMREEKSPNDPPLTTEDIKTYYGLIVDECDEMFVDESGKGIHLYSYASI  1280
ABA46383.1   1157  NIRDISFSALEKIRSLKIRQFNYKNAVNELYRMREEKSPNDPPLTTEDIKTYYGLIVDECDEMFVDESGKGIHLYSYASI  1236
YP_338198.1  1157  NIRDISFSALEKIRSLKIRQFNYKNAVNELYRMREEKSPNDPPLTTEDIKTYYGLIVDECDEMFVDESGKGIHLYSYASI  1236
YP_512324.1  1157  NIRDISFSALEKIRSLKIRQFNYKNAVNELYRMREEKSPNDPPLTTEDIKTYYGLIVDECDEMFVDESGKGIHLYSYASI  1236

QOQ37199.1   1259  GIKGLQEVDEEVQEQKVEIANLKSQVASQENRIAQLEE-LLQQLIDKKPEQP  1309
QOQ37255.1   1257  TVKALQEVDATVQEQEGEIANLKSQVASQEDRIARLEELLLQQLIDKKPEQP  1308
ARW58514.1   1270  TVKALQEVDATVQEQEGEIANLKSQVASQEDRIARLEELLLQQLIDKKPEQP  1321
ARW58396.1   1281  GIKGLQEVDATVQEQEVEIANLKSQIASQEDRIARLEELLLQKLINEKPEQP  1332
AZF88360.1   1281  GIKGLQEVDATVQEQEVEIENLKSKVASQEDRIARLEELLLQRLINKKPEQP  1332
YP_459979.1  1280  GIKGLQEVDATVQEQEVEIANLKSQIASQEDRIARLEELLLQQLINKKPEQP  1331
QOQ37148.1   1304  GIKGLQEVDATVQEQEVEIENLKSQVASQEDRIARLEELLLQQLIDKKPEQP  1355
ARW58457.1   1281  GIKGLQEVDATVQEQEVEIANLKSQIASQEDRIARLEELLLQKLINKKPEQL  1332
ABA46383.1   1237  GIKGLQEVDATVQEQEVEIANLKSQIASQEDRIARLEELLLQQLINKKPEQP  1288
YP_338198.1  1237  GIKGLQEVDATVQEQEVEIANLKSQIASQEDRIARLEELLLQQLINKKPEQP  1288

YP_512324.1  1237  GIKGLQEVDATVQEQEVEIANLKSQIASQEDRIARLEELLLQQLINKKPEQP  1288
```

Figure S3. Alignment of amino acid sequences of Tal/RBP proteins (J5a_015 and its counterparts) of J5a, F16Ba, z1a, and other Wbeta-like bacteriophages.
